# Thermoelectric Silver‐Based Chalcogenides

**DOI:** 10.1002/advs.202204624

**Published:** 2022-10-26

**Authors:** Si Yin Tee, Daniel Ponsford, Chee Leng Lay, Xiaobai Wang, Xizu Wang, Darren Chi Jin Neo, Tianze Wu, Warintorn Thitsartarn, Jayven Chee Chuan Yeo, Guijian Guan, Tung‐Chun Lee, Ming‐Yong Han

**Affiliations:** ^1^ Institute of Materials Research and Engineering Singapore 138634 Singapore; ^2^ Department of Chemistry University College London London WC1H 0AJ UK; ^3^ Institute for Materials Discovery University College London London WC1E 7JE UK; ^4^ Institute of Sustainability for Chemicals Energy and Environment Singapore 627833 Singapore; ^5^ Institute of Molecular Plus Tianjin University Tianjin 300072 China

**Keywords:** multinary alloys, near‐room‐temperature thermoelectric materials, silver‐based chalcogenides, thermal energy harvesting, waste heat recovery

## Abstract

Heat is abundantly available from various sources including solar irradiation, geothermal energy, industrial processes, automobile exhausts, and from the human body and other living beings. However, these heat sources are often overlooked despite their abundance, and their potential applications remain underdeveloped. In recent years, important progress has been made in the development of high‐performance thermoelectric materials, which have been extensively studied at medium and high temperatures, but less so at near room temperature. Silver‐based chalcogenides have gained much attention as near room temperature thermoelectric materials, and they are anticipated to catalyze tremendous growth in energy harvesting for advancing internet of things appliances, self‐powered wearable medical systems, and self‐powered wearable intelligent devices. This review encompasses the recent advancements of thermoelectric silver‐based chalcogenides including binary and multinary compounds, as well as their hybrids and composites. Emphasis is placed on strategic approaches which improve the value of the figure of merit for better thermoelectric performance at near room temperature via engineering material size, shape, composition, bandgap, etc. This review also describes the potential of thermoelectric materials for applications including self‐powering wearable devices created by different approaches. Lastly, the underlying challenges and perspectives on the future development of thermoelectric materials are discussed.

## Graphical abstract

Silver‐based chalcogenides are promising room temperature thermoelectric materials, which are anticipated to catalyze tremendous progress in energy harvesting. This comprehensive review encompasses recent advancements of their binary and multinary (ternary, quaternary, quinary, senary, and septenary) compounds in doped, alloyed, hybridized and composited forms, for a vast range of applications.

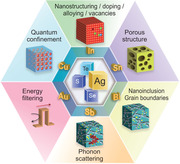

## 1 Introduction

The concept of thermoelectricity dates from 1821, with the discovery of the Seebeck effect by T. J. Seebeck. This effect describes the creation of a voltage which arises from the temperature difference between two points of an electrically conducting material.^[^ ^1^ ^]^ In 1834, J. C. A. Peltier discovered the analogous reverse effect by generating heat flow with an electric current. Two decades later in 1854, W. Thomson demonstrated that reversible heating or cooling was possible under an electric current and in the presence of a temperature gradient. The combination of the Seebeck, Peltier, and Thomson effects define the field of thermoelectrics today, although it took almost a century for the initial discoveries to translate into an active field of research.^[^ ^2^ ^]^ In the early 20^th^ century, thermoelectric materials were actively studied for use in valuable technologies, particularly for cooling and power generation applications in both civilian and military scenarios.

A. F. Ioffe developed the modern theory of semiconductor physics and enabled its applicability to thermoelectrics in 1949 by introducing the thermoelectric figure of merit, *zT = S* ^2^ *σT/κ*, to evaluate thermoelectric performance, where *S* is the Seebeck coefficient, *σ* is the electrical conductivity, *κ* is the thermal conductivity, and *T* is the absolute temperature.^[^ ^3^ ^]^ It was found that thermoelectric materials with a high *zT* are typically heavily doped semiconductors, the best‐known examples being the tellurides of antimony, bismuth, and lead. In the 1950s, Goldsmid and Douglas demonstrated thermoelectric cooling to 0 °C with Bi_2_Te_3,_^[^ ^4^ ^]^ the Soviet Union developed the first commercial thermoelectric generator using ZnSb,^[^ ^3^ ^]^ and Chasmar and Stratton established a general strategy to quantify the thermoelectric performance of semiconductors depending on their effective mass, carrier mobility, doping, temperature, and thermal conductivity.^[^ ^5^ ^]^ In 1995, Slack introduced a phonon‐glass/electron‐crystal strategy to disrupt phonon transport without affecting electron transport, opening up a new avenue for engineering thermoelectric materials with complex structures such as clathrates and skutterudites, which have large voids in their crystal structures. These developments have significantly broadened the commercial usage of devices capable of converting waste heat to electricity.^[^ ^6^ ^]^

The most widely investigated thermoelectric materials to date are metal chalcogenides based on Bi–Te/Sn–Se/Cu–Se, half‐Heusler compounds, multicomponent oxides, GeTe/PbTe hybrids, and organic‐inorganic composites.^[^ ^7^ ^]^ Thermoelectric performance is generally assessed at low temperature (<300 K), near room temperature (300–500 K), medium temperature (500–800 K) and high temperature (>800 K). Bi–Te‐based materials are perhaps those most commonly utilized with an optimal operation at near room temperature,^[^ ^8^, ^9^ ^]^ whereas (AgSbTe_2_)_1‐_ *_x_* (GeTe)*_x_* are considered as the most prominent materials for applications at medium temperatures.^[^ ^10^, ^11^ ^]^ SiGe is classically used in thermoelectric devices expected to operate at higher temperatures.^[^ ^12^, ^13^ ^]^

Statistical data reveal that low‐grade waste heat with temperatures of <100 °C (373 K) accounts for ≈63% of total waste heat worldwide.^[^ ^14^, ^15^, ^16^ ^]^ Recent developments in room temperature thermoelectric materials promise to go some way to addressing this energy wastage problem. In particular, the excellent near room temperature thermoelectric performance of binary Ag_2_E (E = S, Se, Te) compounds and their multinary derivatives and hybrids is notable. These properties are attributed to the narrow bandgap, high electron mobility, and low electron effective mass required to achieve high electrical conductivity as well as relatively high Seebeck coefficient of such materials.^[^ ^17^ ^]^ Moreover, Ag–Se‐based materials are a desirable alternative to traditional Bi–Te materials because of the scarce availability of tellurium (0.001 ppm) in the Earth's crust^[^ ^18^, ^19^ ^]^ and the toxicity of Bi–Te alloys.

**Figure 1:**
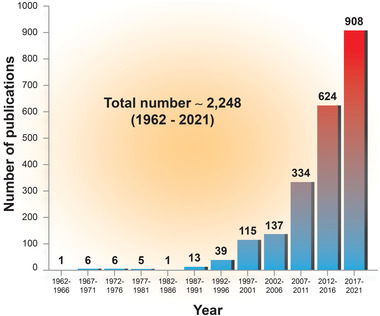
Number of publications in a quadrennial period, as reported by the Web of Science when using the topic “silver thermoelectric” in the search engine.

**Figure 2:**
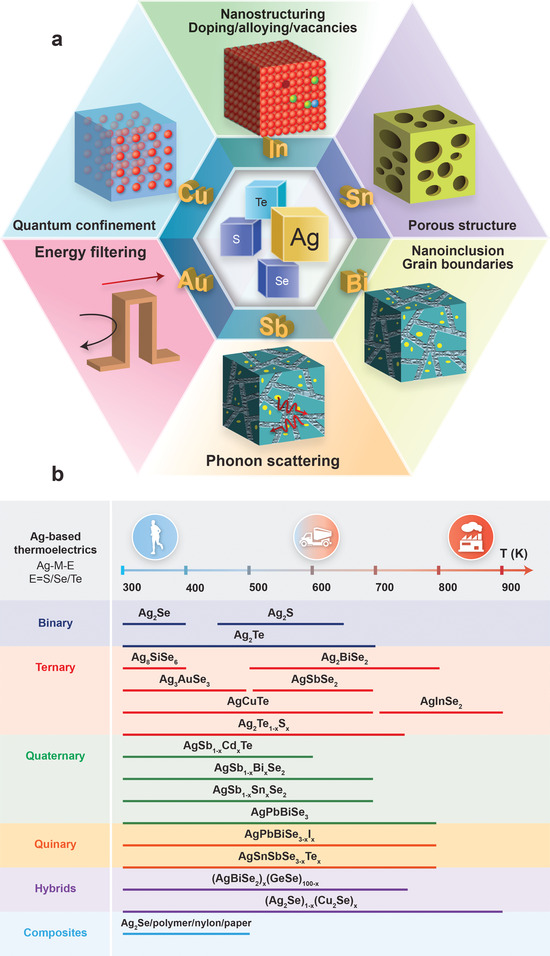
a) Schematic illustration of engineering strategies for optimizing silver‐based chalcogenide thermoelectric materials. b) Schematic comparison of various binary, ternary, quaternary, hybrid, and composite silver‐based chalcogenide thermoelectric materials for the applications of waste heat harvesting and their temperature range of operation.

Most recently, there has been a surge in research articles reporting on the development of silver‐based chalcogenide thermoelectric materials, devices, and applications (**Figure** **1**). This review aims to present a comprehensive survey of the developments in binary, multinary silver‐based chalcogenides, as well as their hybrids and composites (**Table** **1**). First, we introduce the preparative methodologies for silver‐based chalcogenide materials and devices. Secondly, we review the advanced properties and aspects of structural design required for improving thermoelectric performance, by considering quantum confinement, nanostructuring/doping/alloying/vacancies, point defects/dislocations/interfaces/inclusions, and porosity (**Figure** **2**a). Thirdly, we summarize the promising applications of these materials, including the development of flexible thermoelectric materials for wearable devices (Figure 2b). Last, we provide insights and perspectives on the work needed to leverage current successes and address future challenges in the field of silver‐based chalcogenide thermoelectric materials.

**Table 1:** Summary of material preparation and thermoelectric characteristics of silver‐based chalcogenides

Type	Silver‐based chalcogenides	Preparation methods (HP, CP, SPS, LBL, DC, BM)^a)^	*σ* [S cm^−1^]	*S* [µV K^−1^]	*κ* [W m^−1^ K^−1^]	Thermoelectrics (*zT*, PF, PD, *V*, *P*, *V*_oc_)^a)^	Refs.
Binary Ag_2_S, Ag_2_Se, Ag_2_Te	Ag_1.96_S	High pressure preparation	240	–150	0.45	*zT* 0.62, 560 K	[29]
	Ag_2_Se	Melt alloying	1988	–133	11.0	*zT* 0.96, 300 K	[67]
	Ag_2_Se	Melt alloying	714 to 2500	–90 to‐160	0.8 to 2	*zT* 1.0, 300 to 375 K	[19]
	Ag_2_Se	Liquid‐phase sintering	2000	–130	1.2	*zT* 1.21, 389 K	[68]
	Ag_2_Se	Hydrothermal + SPS	1500	–120	1.1	*zT* 0.6, 300 K	[69]
	Ag_2_Se	Aqueous synthesis + SPS	981	–146	0.77	*zT* 0.8, 300 K	[43]
	Ag_1.975_Se_1.025_	Mechanical alloying + pulse discharge sintering	1000	–145	0.8396	*zT* 0.6, 300 K	[37]
	Ag_2.0006_Se	Melt alloying + BM + HP	1430	–135	1.3	*zT* 0.6, 300 K	[70]
	Ag_2_Se_1.06_	Melt alloying + SPS	1290	–153	1.08	*zT* 0.84, 300 K	[71]
	Ag_2_Se_1.02_	Melt alloying + SPS	1200	–130	1.0	*zT* 0.8, 300 K	[72]
	Ag_2_Se	Manual mixing + SPS	1000	–150	1.02	*zT* 0.8, 390 K	[73]
	Ag_2_Se	Manual mixing + CP	1700	–132.5	0.9	*zT* 1.2, 390 K	[74]
	Ag_2_Se	Aqueous synthesis + SPS	6600	–20	2.4	*zT* 0.45, 323 K	[51]
	Ag_2_Se	Melt alloying + Zone melting	1000	–140	0.9	*zT* 0.75, 300 K	[39]
	Ag_2_Se	Wet mechanical alloying + SPS	900, 300K	–150, 300 K	0.83, 300 K	*zT* 0.7, 300 K; *zT* 0.9, 390 K	[75]
	Ag_2_Se	Cationic exchange	750	–90	0.53	*zT* 0.46, 300 K	[57]
	Ag_2_Se	Melt alloying + SPS + pulsed laser deposition	1000	–120	–	PF 1750 µW m^−1^ K^−2^, 300 K	[64]
	Ag_2_Te	Colloidal synthesis +LBL dip coating	13.5	–110	0.045	*zT* 0.32, 390 K	[76]
	Ag_2_Te	Solvothermal + SPS	400	–135	0.53	*zT* 0.9, 623 K	[44]
	Ag:Ag_2_Te	Solvothermal + SPS	400	–140	0.5	*zT* 1.1, 623 K	[44]
	S:Ag_2_Te	Solvothermal + HP	819.9	–150	0.4	*zT* 0.62, 550 K	[77]
Ternary Ag‐E1‐E2 E = S, Se, Te	Ag_2_S_0.5_Se_0.5_	Melt alloying + SPS	300	–140	0.57	*zT* 0.26, 300 K	[20]
	Ag_2_S_0.4_Se_0.6_	Melt alloying + HP	2000	–178	0.7	*zT* 1.08, 350 K	[78]
	Ag_4_SeS	Colloidal synthesis + HP	395	–129	0.71	*zT* 0.33, 355 K	[45]
	Ag_2_S_0.4_Te_0.6_	Melt alloying + HP	800	–83	0.78	*zT* 0.2, 300 K	[79]
	Ag_2_S_0.4_Te_0.6_	Melt alloying + HP	500	–120	0.63	*zT* 0.7, 573 K	[79]
	Ag_2_S_0.8_Te_0.2_	Melt alloying + SPS	150	–145	0.33	*zT* 0.63, 450 K	[20]
	Ag_2_S_0.8_Te_0.2_	Melt alloying + SPS	250	–150	0.44	*zT* 0.63, 450 K	[20]
	Ag_20_S_7_Te_3_	Melt alloying	230	–175	0.5	*zT* 0.8, 600 K *V*_OC_ 69.2 mV, *P* 17.1 µW, ΔT 70 K	[80]
Ternary Ag‐M‐S/Se/Te M = Si, K, Cu, Ga, In, Sn, Sb, Au, Bi	KAg_3_Se_2_	Bridgman method	0.1, RT	–	0.4, RT	–	[21]
	Ag_1.9_Sn_0.1_Se	Chemical synthesis + SPS	2130	–115	0.88	*zT* 0.9, 300 K	[81]
	Ag_1.98_Cu_0.02_Se	Aqueous synthesis + SPS	1778	–120.1	0.83	*zT* 1.2, 393 K	[43]
	Ag_3_AuSe_2_	Colloidal synthesis + HP	900	–130	0.6	*zT* 0.88, 390 K	[46]
	Ag_8_SiSe_6_	Melt alloying + HP	1000	–130	0.9	*zT* 0.6, 300K	[82]
	AgSb_0.99_Se_2_	Melt alloying	75	300	0.4	*zT* 1, 610 K	[83]
	AgInSe_2_	Melt alloying + HP	80	–295	0.39	*zT* 1.1, 900 K	[84]
	AgCuSe	Melt alloying + SPS	1200	–118	1.01	*zT* 0.6, 450 K	[85]
	AgCuSe	Aqueous synthesis + SPS	1050	–90	0.7	*zT* 0.42, 323 K	[86]
	AgCuSe	Aqueous synthesis + SPS	100	210	0.3	*zT* 0.9, 623 K	[86]
	AgCuSe	Colloidal synthesis + HP	100	–190	0.25	*zT* 0.68, 566 K	[87]
	AgCuTe	Melt alloying	150	240	0.4	*zT* 1.45, 700 K	[88]
	AgCuS	Chemical synthesis + CP + sintering	1.8	400	0.55	*zT* 0.03, 380 K	[89]
	AgBiS_2_	Chemical synthesis + CP + sintering	32	–195	0.55	*zT* 0.2, 810 K	[90]
	AgBiS_2_	Melt alloying + SPS	100	–235	0.48	*zT* 0.7, 823 K	[91]
	Ag_0.85_InTe_2_	Melt alloying + SPS	5	375	0.1	*zT* 0.62, 814 K	[92]
	AgGa_0.93_Te_2_	Melt alloying + SPS	13	400	0.2	*zT* 1, 873 K	[93]
Quaternary Ag‐M1‐M2‐S/Se/Te M = Na, Mg, Ca, Mn, Ni, Cu, Zn, Nb, Cd, Ga, In, Sn, Sb, Ba, Pb, Bi	AgCuSe_0.95_Te_0.05_	Melt alloying + SPS	1000	–115	0.8	*zT* 0.7, 450 K	[94]
	AgCuSe_0.1_Te_0.9_	Melt alloying	200	225	0.5	*zT* 1.6, 700 K	[88]
	(AgCu)_0.995_Se_0.1_Te_0.9_	Mechanical alloying + BM + HP	25	240	0.45	*zT* 1.1, 350 K	[95]
	Ag_2_S_0.4_(Se_0.6_Te_0.4_)_0.6_ + 0.04Se	Melt alloying	900	–85	0.7	*zT* 0.3, 300 K	[96]
	AgBiSe_1.98_Cl_0.2_	Melt alloying	190	–165	0.5	*zT* 0.9, 805 K	[97]
	AgCu_0·96_Ni_0·04_Se	Melt alloying + HP	100	–200	0.4	*zT* 0.8, 623 K	[22]
	AgSb_0.99_Na_0.01_Se_2_	Melt alloying + HP	44	340	0.37	*zT* 0.92, 673 K	[98]
	AgSb_0.96_Pb_0.04_Se_2_	Melt alloying	20	400	0.3	*zT* 0.7, 680 K	[99]
	AgSb_0.98_Ca_0.02_Se_2_	Mechanical alloying + HP	1000	260	0.37	*zT* 1.2, 673 K	[100]
	AgSb_0.96_Mn_0.04_Se_2_	Melt alloying + SPS	84	275	0.44	*zT* 1.05, 673 K	[101]
	AgSb_0.99_Sn_0.01_Se_2_	Melt alloying + HP	100	295	0.43	*zT* 1.21, 660 K	[102]
	AgSb_0.98_Mg_0.02_Se_2_	Melt alloying + SPS	67	300	0.40	*zT* 1, 673 K	[103]
	AgSb_0.98_Ba_0.02_Se_2_	Melt alloying + SPS	40	360	0.35	*zT* 1, 673 K	[103]
	AgSb_0.98_Bi_0.02_Se_2_	Melt alloying	35	375	0.35	*zT* 1.15, 680 K	[99]
	AgSb_0.98_Bi_0.02_Se_2_	Colloidal synthesis + HP	60	350	0.4	*zT* 1.1, 640 K	[104]
	AgSb_0.98_Cd_0.02_Se_2_	Melt alloying	53	325	0.38	*zT* 1, 640 K	[105]
	AgSb_0.94_Cd_0.06_Se_2_	Melt alloying + SPS	250	260	0.4	*zT* 2.6, 573 K	[106]
	Ag_0.96_Nb_0.04_BiSe_2_	Melt alloying + SPS	200	–215	0.7	*zT* 1, 773 K	[107]
	Ag_0.985_In_0.015_BiSe_2_	Melt alloying + SPS	125	–185	0.5	*zT* 0.7, 773 K	[108]
	AgPbBiSe_3_	Melt alloying	70	–195	0.52	*zT* 0.43, 818 K	[109]
	Ag_8.3_Cu_0.7_GaSe_6_	Melt alloying + HP + annealing	200	–200	0.45	*zT* 1.4, 800 K	[110]
	(Ag_0.2_Cu_0.785_)_2_S_0.7_Se_0.3_	Melt alloying	100	275	0.65	*zT* 0.95, 800 K	[111]
	AgSb_0.94_Cd_0.06_Te_2_	Melt alloying	250	264	0.4	*zT* 2.6, 573 K	[106]
	AgSb_0.96_Zn_0.04_Te_2_	Melt alloying	210	290	0.55	*zT* 1.9, 584 K	[112]
	Ag_0.2_Cu_0.89_In_0.91_Te	Melt alloying + SPS	80	325	0.47	*zT* 1.6, 850 K	[113]
	AgMnSbTe_3_	Melt alloying + SPS	242	225	0.7	*zT* 1.46, 823 K	[114]
	Ag_0.025_In_0.025_SnTe_1.05_	Melt alloying	1100	170	2.75	*zT* 1, 856 K	[115]
Other multinary: quinary, senary and septenary	AgSnSbSe_1.5_Te_1.5_	Melt alloying + SPS	260	185	0.64	*zT* 1.14, 723 K	[116]
	AgPbBiSe_2.97_I_0.03_	Melt alloying	115	–190	0.4	*zT* 0.8, 814 K	[109]
	Ag_0.2_Cu_0.8_In_0.2_Ga_0.8_Te_2_	Melt alloying + SPS	250	140	0.7	*zT* 1.5, 850 K	[117]
	(AgCu)_0.998_Se_0.22_S_0.08_Te_0.7_	Melt alloying	68	260	0.25	*zT* 0.68, 340 K	[118]
	(AgCu)_0.998_Se_0.22_S_0.08_Te_0.7_/Ag_20_S_7_Te_3_	Melt alloying	–	–	–	*V*_OC_ 0.2 mV, *P* 70 nW, PD 11 µW cm^−2^ K^−2^ at 298 K on human's wrist	[118]
	Ag_0.1_Cu_1.9_Te_0.6_S_0.2_Se_0.2_	Melt alloying	110	220	0.7	*zT* 1.4, 1000 K	[119]
	Ge_0.62_Ag_0.11_Sb_0.13_Pb_0.12_Te	Melt alloying + SPS	700	200	1.0	*zT* 2.5, 750 K	[120]
	Ge_0.61_Ag_0.11_Sb_0.13_Pb_0.12_Bi_0.01_Te	Melt alloying + SPS	600	225	0.8	*zT* 2.7, 750 K	[120]
	Ge_0.56_Ag_0.11_Sb_0.13_Pb_0.12_Bi_0.01_Mn_0.05_Te	Melt alloying + SPS	500	225	0.75	*zT* 2.7, 750 K	[120]
	Ge_0.56_Ag_0.11_Sb_0.13_Pb_0.12_Bi_0.01_Sn_0.05_Te	Melt alloying + SPS	450	230	0.75	*zT* 2.5, 750 K	[120]
Multinary hybrids	(AgBiSe_2_)_0.97_(GeSe)_0.03_	Bridgman method	330	–105	0.25	*zT* 1.05, 748 K	[40]
	(AgSbSe_2_)_20_(GeTe)_80_	Melt alloying	500	250	0.8	*zT* 1.9, 660 K	[121]
	(AgBiSe_2_)_20_(GeTe)_80_	Melt alloying	150	275	0.5	*zT* 1.3, 467 K	[122]
	(AgBiSe_1.995_Br_0.005_)_50_ (GeTe)_50_	Melt alloying	110	–175	0.25	*zT* 0.6, 500 K	[122]
	(AgBiSe_2_)_0.50_(GeSe)_0.50_	Melt alloying	120	–170	0.6	*zT* 0.45, 677 K	[123]
	(AgBiSe_2_)_0.22_(SnSe)_0.78_	Melt alloying + BM + SPS	675	75	0.6	*zT* 1.3, 823 K	[124]
	AgSbSe_2_/ZnSe (2 mol%)	Melt alloying	70	290	0.38	*zT* 1.1, 635 K	[125]
	(AgSbSe_2_)_0.5_(SnSe)_0.5_	Melt alloying	80	240	0.5	*zT* 0.77, 725 K	[126]
	(AgSb_0.94_Ge_0.06_Se_2_)_0.5_ (SnSe)_0.5_	Melt alloying	150	225	0.54	*zT* 1.05, 706 K	[126]
	(Ag_2_Se)_0.95_(Cu_2_Se)_0.05_	Melt alloying/micro‐wave‐assisted thermolysis + SPS	1400	–70	1.0	*zT* 0.45, 875 K	[54]
	AgInSe_2_/Ag_2_Se	Melt alloying + SPS	50	–200	–	*zT* 0.9, 846 K	[127]
	1%Br:(AgBiSe_2_)_0.7_(PbSe)_0.3_	Melt alloying + HP	150	–170	0.45	*zT* 0.8, 800 K	[128]
	Ag_2_Se/5 mol%Te	Colloidal synthesis + HP	1000	–165	0.7	*zT* up to 0.79, RT	[17]
Ag_2_Se composites	Ag_2_Se/glass	Pulsed hybrid reactive magnetron sputtering	800	–175	0.43	*zT* 1.2, RT	[66]
	Ag_2_Se/nylon	Chemical synthesis + HP	497	–140	0.449	PF 987.4 µW m^−1^ K^−2^, 300 K PD 2.3 W m^−2^, Δ*T* 30 K	[47]
	Ag_2_Se/nylon	Chemical synthesis + HP	920	–143	0.696	PF 1882 µW m^−1^ K^−2^, 300 K PD 22 W m^−2^, Δ*T* 30 K	[129]
	Ag/Ag_2_Se/nylon	Chemical synthesis + HP	3958	–67.5	–	PF 1860.6 µWm^−1^ K^−2^, 300 K	[130]
	Ag/Ag_2_Se/nylon	Microwave‐assisted synthesis + HP	3030	–90	–	PF 2436 µW m^−1^ K^−2^, 300 K, *V* 16.1 mV; *P* 6.08 µW, ΔT 29.6 K	[131]
	Ag_2_Se/Ag/CuAgS/nylon	Chemical synthesis + HP	11000	–45	–	PF 2231.5 mW m^−1^ K^−2^, 300 K PD 5.42 W m^−2^, ΔT 45 K	[132]
	PVP‐Ag_2_Se/nylon	Chemical synthesis + HP	929	–143	–	PF 1910 µW m^−1^ K^−2^, *zT* 1.1, 300 K PD 28.8 W m^−2^, Δ*T* 29.1 K	[133]
	PPy‐Ag_2_Se/Se/nylon	Chemical synthesis + HP	1064	–144	–	PF 2240 µW m^−1^ K^−2^, 300 K PD 37.6 W m^−2^, Δ*T* 34.1 K	[134]
	PANI‐Ag_2_Se/PVDF	Chemical synthesis + DC	268	–85.5	–	PF 196.6 µW m^−1^ K^−2^, 300 K *V* 15.4 mV, *P* 835.8 nW, ∆T 30 K	[135]
	Ag_2_Se/PVDF	Chemical synthesis + DC	320	–75	–	PF 180.6 mW m^−1^ K^−2^, 400 K	[136]
	Ag_2_Se/PVDF	Aqueous synthesis + CP	205.52	–95.9	7.0, in‐plane	PF 189.02 µW m^−1^ K^−2^; *zT* 0.0079, 300 K; *V* 5.86 mV; *P* 4.9 nW, ΔT 30 K	[137]
	Ag/Ag_2_Se/PEDOT/nylon	Chemical synthesis + HP	5957.3	–50	–	PF 1442.5 µW m^−1^ K^−2^, 300 K PD 7.47 W m^−2^, *P* 1.8 µW, ΔT 27 K	[138]
	Ag_2_Se/PEDOT:PSS	Chemical synthesis + DC	660	–52	–	PF 178.59 µW m^−1^ K^−2^, 300 K	[139]
	Ag_2_Se/paper	Solvothermal synthesis + CP	1660	–120	–	PF 2450.9 µW m^−1^ K^−2^, 303 K	[140]
	Ag_2_Se/photoresin	Vat photopolymerization	100	–72	0.13	PF 51.5 µW m^−1^ K^−2^, *zT* 0.12, 300 K	[31]
	Ag_2_Se/photoresin/PEDOT:PSS	Vat photopolymerization	120	–84	1.13	*zT* 0.02, 300 K	[32]
	Ag_2_Se‐based printed film	Screen printing + HP	–60	–0.25	0.3	PF 540 µW m^−1^ K^−2^, *zT* 0.6, 323 K PD 321 µW cm^−2^, ΔT 110 K *V* 72.2 mV, ΔT ≈ 30 K	[141]
	Ag_2_Se‐based printed film	Screen printing + HP	460	–190	0.5, in‐plane	PF 17 µW cm^−1^ K^−2^, *zT* 1.03, 300 K *V* 17.6 mV, *P* 0.19 µW, ΔT 75 K	[142]
	Ag_2_Se‐based printed film	Screen printing + HP	472	–183	0.48	*zT* 1, 300 K	[143]
	Ag_2_Se/3D scaffolds	Mixing and stirring	–	–	–	*V*_OC_ 4.2 mV at RT on body skin	[143]
Ag_2_Te composites	Ag_2_Te/paper	Hydrothermal synthesis + CP	95.3	–142	–	PF 192 µW m^−1^ K^−2^, 195 °C	[144]
	Ag_2_Te/paper	Hydrothermal synthesis + vacuum filtration/DC	153.35	–99.48	–	PF 151.76 µW m^−1^ K^−2^, 300 K	[145]
	Ag_2_Te/P(NDI2OD‐T2)	Chemical synthesis + DC	0.61	–130	–	PF ≈ 1 µW m^−1^ K^−2^, 300 K	[146]
	Ag_2_Te/PVDF	Chemical synthesis + DC	86	–60	–	PF 30 µW m^−1^ K^−2^, 300 K	[147]
	Ag_2_Te/PEDOT:PSS	Aqueous synthesis + DC	–	100	–	*V* 2.6 mV, ΔT 298 K	[148]

^a)^ *zT*: figure of merit; PF: power factor; PD: power density; *V*: output voltage; *P*: output power; *V*_OC_: open circuit voltage; Δ*T*: temperature difference; SPS: spark plasma sintering; HP: hot‐pressing; CP: cold‐pressing; LBL: layer‐by‐layer; DC: drop casting; BM:ball milling.

## 2 Preparative Methodologies in Solid, Liquid, and Vapor Phases

**Figure 3:**
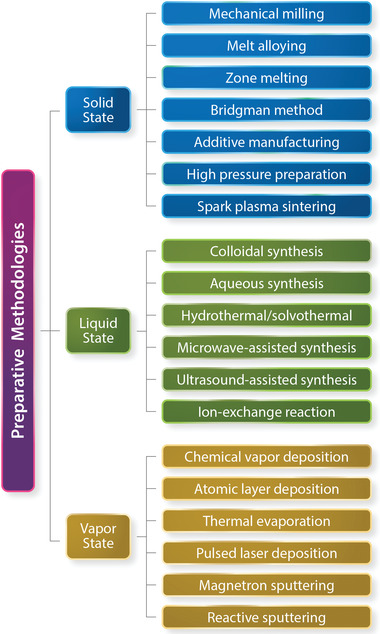
Preparative methodologies of silver‐based chalcogenides based on solid‐, liquid‐, and vapor‐state reaction. Solid‐state reaction methods include mechanical milling, melt alloying, zone melting, Bridgman method, additive manufacturing, high‐pressure preparation, and spark plasma sintering. Liquid‐state reaction preparations usually involve wet‐chemical routes in aqueous solutions/organic solvents through colloidal synthesis, hydrothermal/solvothermal precipitation, microwave‐/ultrasound‐assisted preparation or template‐assisted ion exchange reactions. Vapor‐state reactions encompass a variety of physical and chemical vapor deposition techniques.

Silver‐based chalcogenides have been prepared via solid‐state, liquid‐state, and vapor‐state reactions (**Figure** **3**). The solid‐state reaction methods include mechanical alloying, melt alloying, additive manufacturing, and crystal growth such as the Bridgman method or zone melting. The liquid‐state reaction routes (also known as wet‐chemical syntheses) comprise colloidal synthesis, hydrothermal or solvothermal precipitation, microwave‐ or ultrasound‐assisted preparation, and template‐assisted ion exchange reactions. The vapor‐state preparation approaches encompass a variety of physical and chemical vapor deposition techniques.

### 2.1 Solid‐State Preparation

Thermoelectric bulk materials can be prepared by blending two or more elements in stoichiometric quantities and then heating at a high temperature (500–2000 °C) in sealed quartz ampules. These solid‐state reactions require a significant amount of heat to overcome the lattice energy, and enable the diffusion of cations and anions into various sites in products including Ag_2_S,^[^ ^20^ ^]^ Ag_2_Se,^[^ ^19^ ^]^ Ag_2_S_0.8_Te_0.2_,^[^ ^20^ ^]^ KAg_3_Se_2,_^[^ ^21^ ^]^ and AgCu_0·96_Ni_0·04_Se.^[^ ^22^ ^]^ This traditional process is time‐consuming and energy‐intensive, and also presents difficulties for large‐scale production. Meanwhile, the presence of trace impurities or secondary phases in products will greatly affect their thermoelectric properties. These effects arise when reactants are not present in exact stoichiometric proportions, and if unreacted residues remain following the reaction.^[^ ^23^ ^]^ As the microstructures and properties of solidified ingots are strongly related to their liquid states,^[^ ^24^ ^]^ liquid‐state sintering has been used to effectively optimize the microstructures to improve thermoelectric performance. This sintering method enables the rearrangement, dissolution, and reprecipitation of grains.^[^ ^25^, ^26^, ^27^ ^]^ Compared to conventional preparation routes of silver chalcogenides, a high‐pressure preparation method (≈1–5 GPa) has been developed, which aims to prevent the loss of volatile raw materials via sublimation, by reducing reaction temperature and shortening reaction.^[^ ^28^, ^29^ ^]^

Additive manufacturing, otherwise known as 3D printing, is a technique which can be used to construct a vast range of materials with complex geometries and layered structures. Thermoelectric materials created by additive manufacturing are particularly novel and the field remains in its infancy.^[^ ^30^ ^]^ The various additive manufacturing techniques include vat photopolymerization, powder bed fusion processes, material jetting techniques, and extrusion‐based methods. Vat photopolymerization has been successfully employed for the fabrication of room‐temperature thermoelectric polymer composites comprising *β*‐Ag_2_Se.^[^ ^31^, ^32^ ^]^ Photopolymerization‐based processes can print high‐quality surface finishes at a fine resolution. However, the drawbacks of this technique include a restricted choice of photoresists and a complex curing process.^[^ ^33^ ^]^ Powder bed fusion techniques employ selective laser melting and selective laser sintering technology that uses a heat source, such as a high power‐density laser, to melt and fuse regions of a powder bed,^[^ ^34^ ^]^ while material jetting provides a means to deposit materials in liquid or solid suspensions.^[^ ^35^ ^]^ Owing to the simplicity of the process, the extrusion of inks containing inorganic particles is one of the most widely utilized printing techniques for the production of inorganic thermoelectric materials with precise shape and dimension.^[^ ^36^ ^]^

Mechanical alloying (under wet or dry conditions) is commonly used to produce nano/microstructured silver‐based chalcogenides with reduced thermal conductivities.^[^ ^37^, ^38^ ^]^ A drawback of this technique is the extended and energy‐intensive milling process, which is required. Zone melting can directly generate bulk Ag_2_Se polycrystals without the need for additional sintering processes, thus avoiding possible elemental inhomogeneity and the production of off‐stoichiometric silver precipitates or metastable phases, which arise from the migration of Ag^+^ and the volatilization of Se at high temperatures.^[^ ^39^ ^]^ Alternatively, the Bridgman method is used to grow AgBiSe_2_ and Ge‐doped (GeSe)*_x_* (AgBiSe_2_)_1−_ *_x_* through directional solidification, by translating a melt from the hot zone to the cold zone in furnace.^[^ ^40^ ^]^ As reported, Bridgman‐grown AgBiSe_2_ possesses a low electrical resistivity, likely ascribed to high packing density, high crystallinity and presence of fewer grain boundaries.

### 2.2 Liquid‐State Preparation

Providing enhanced control of stoichiometry, structure, and phase purity, wet‐chemical syntheses in the liquid phase have become an increasingly important way to prepare a variety of metal chalcogenides,^[^ ^41^ ^]^ and their nanostructured derivatives.^[^ ^42^ ^]^ These syntheses present an energy‐efficient strategy to synthesize a wide variety of binary (Ag_2_Se,^[^ ^43^ ^]^ Ag_2_Te^[^ ^44^ ^]^) and ternary (Ag_4_SeS,^[^ ^45^ ^]^ Ag_3_AuSe_2_^[^ ^46^ ^]^) silver‐based chalcogenides and their hybrids or composites.^[^ ^47^ ^]^ In organic solvents, the colloidal synthesis of organic‐coated chalcogenides often involves the thermal decomposition of precursors in the presence of surfactants,^[^ ^45^, ^48^, ^49^, ^50^ ^]^ which is unfavorable due to reduced electrical conductivity and usually requires a prior ligand displacement procedure.^[^ ^17^ ^]^ Aqueous synthesis is preferred for the large‐scale production of chalcogenides (e.g., binary Ag_2_Se, hybrid Ag^0^:Ag_2_Se and ternary Cu^+^:Ag_2_Se) at room temperature and atmospheric pressure, as high temperatures and organic solvents/surfactants are not required.^[^ ^51^ ^]^

Hydrothermal/solvothermal syntheses proceed in aqueous/non‐aqueous solutions in closed stainless steel autoclaves at high temperature and pressure.^[^ ^44^, ^51^, ^52^ ^]^ Microwave‐assisted syntheses are carried out in solution and are enabled by the friction and collisions between polar molecules under an alternating electromagnetic field.^[^ ^53^, ^54^, ^55^ ^]^ Alternatively, sonochemistry is used to produce inorganic particles via chemical reaction under powerful ultrasound radiation between 20 kHz and 15 MHz.^[^ ^56^ ^]^ Ion exchange reactions are commonly used to synthesize semiconductor nanocrystals, whereby guest cations are able to substitute with the host cations within the crystal lattice. A successful example of a cationic exchange transformation is the fabrication of polycrystalline Ag_2_Se thin films, which was achieved by soaking Cu_2‐_ *_x_* Se films in a Ag^+^‐rich solution.^[^ ^57^ ^]^

### 2.3 Vapor‐Phase Preparation

Vapor condensation and surface reactions enable the formation of highly pure particles from individual atoms or molecules in the gas phase. The vapor‐phase preparation process is highly susceptible to changes in vapor concentration, temperature, and pressure. Chemical and physical vapor phase deposition are two common methods for the fabrication of silver‐based chalcogenide thin films. Chemical vapor deposition (CVD) is the formation of a thin film from the gaseous phase via the reaction and/or decomposition of volatile precursors on a substrate surface. Conventional CVD has been used for the growth of nanowire arrays in thermoelectric microgenerators,^[^ ^58^ ^]^ as well as the fabrication of *β*‐Ag_2_Se with unique hollow and layered branch‐like morphology.^[^ ^59^ ^]^ Various modifications to traditional CVD processes also exist. Plasma‐enhanced CVD utilizes plasma to catalyze the reaction of precursors at lower temperatures. Alternatively, atomic layer deposition is a CVD technique, which harnesses self‐limiting and sequential reactions to produce ultrathin, conformal films with atomic‐scale control over thickness and composition.^[^ ^60^, ^61^ ^]^ However, the atomic layer deposition of metal chalcogenide films is limited by the availability, reactivity, and toxicity of appropriate precursors.^[^ ^62^ ^]^

In comparison, physical vapor deposition (PVD) involves only physical methods to deposit vaporized materials from solid targets onto substrates. For instance, a simple thermal evaporation route was used to prepare thin films of Ag_2_Se with various thicknesses by using a heat source to evaporate bulk polycrystalline Ag_2_Se in vacuo.^[^ ^63^ ^]^ A related technique, pulsed laser deposition, is carried out by focusing a high‐energy pulsed laser beam on a solid target material, such as Ag_2_Se, inside a vacuum chamber. Using this method, the nonepitaxial growth of Ag_2_Se thermoelectric thin films with desired phase and composition has been demonstrated.^[^ ^64^ ^]^ Further iterations of PVD include reactive sputtering, in which compound films are deposited on substrates by introducing a reactive gas precursor, and magnetron sputtering, whereby magnetically confined plasma collides with an electrode or target in order to eject atoms for deposition on a substrate. Recently, pulsed hybrid reactive magnetron sputtering was used to develop a series of high‐performance thermoelectric thin films at room temperature without the requirement for high‐temperature post‐treatment.^[^ ^65^, ^66^ ^]^ Consequently, this method is particularly suitable for fabricating silver chalcogenide thin films with highly accurate stoichiometry on polymer substrates for applications such as wearable devices.^[^ ^66^ ^]^

### 2.4 Densification of Powders

Generally, as‐prepared thermoelectric materials are first sintered and then densified into pellets prior to characterization. The densification process is critically important, since the microstructures formed during the process strongly influence the thermoelectric properties of the material. Applying pressure during the sintering process causes an increase in the driving force and kinetics of the densification. Spark plasma sintering (SPS) has become the most employed densification method, presenting significant advantages over conventional hot‐pressing sintering, such as lower sintering temperatures and pressures, and shorter dwell times. As a result, coarsening and grain growth are minimized, and finer‐grained dense structures and high relative densities can be achieved in a short time. Most importantly, nanosized powders can be sintered in this way without considerable grain growth.^[^ ^23^ ^]^

## 3 Binary Silver Chalcogenides (Ag_2_E)

Binary silver chalcogenides, Ag_2_E (E = S/Se/Te), including silver sulfide, selenide, and telluride have emerged as promising candidate materials for heat energy harvesting at near room temperature (Table 1). Silver oxide (Ag_2_O) has a larger bandgap (2.25 eV) than the rest of the silver chalcogenides (0.06–1 eV) and is not considered to be a good thermoelectric material. The optimal bandgap for maximizing the thermoelectric z𝑇 of a semiconducting material is found to be in the range of 6𝑘_𝐵_𝑇 to 10𝑘*_𝐵_* 𝑇, where 𝑘_𝐵_ is the Boltzmann constant and 𝑇 is the operating temperature in kelvin.^[^ ^149^ ^]^ According to this empirical rule, the narrow bandgaps of Ag_2_S, Ag_2_Se, and Ag_2_Te semiconductors would indicate their potential for good thermoelectric performance.

### 3.1 Silver Sulfide (Ag_2_S)

Ag_2_S exists as three polymorphs: *α*‐Ag_2_S, *β*‐Ag_2_S, and *γ*‐Ag_2_S.^[^ ^150^ ^]^ Monoclinic *α*‐Ag_2_S occurs at temperatures below ≈450 K, while cubic *β*‐Ag_2_S exists in the temperature range 452–859 K, possessing a body‐centered cubic (bcc) sublattice of S^2−^ with superionic properties. The *γ*‐Ag_2_S phase, with a face‐centered cubic (fcc) sublattice of S^2−^, is stable from ≈860 K up to melting temperature.^[^ ^151^ ^]^

Possessing a wider bandgap than Ag_2_Se and Ag_2_Te (≈1 eV),^[^ ^152^ ^]^ Ag_2_S exhibits a low electrical conductivity of approximately 10^−1^ S m^−1^, together with a low Seebeck coefficient and reasonably high electronic thermal conductivity; this combination of properties results in limited thermoelectric performance. To optimize the carrier concentration of Ag_2_S, nonstoichiometric Ag_2−_ *_x_* S was prepared by introducing Ag vacancies which enhance the electrical transport properties and prevent the loss of volatile sulfur through sublimation.^[^ ^29^ ^]^ Furthermore, the high‐pressure approach used in this case promoted the formation of porous structures, which in turn reduced the lattice thermal conductivity, and thus a *zT* of 0.62 at 560 K was reached for the Ag_1.96_S system.

**Figure 4:**
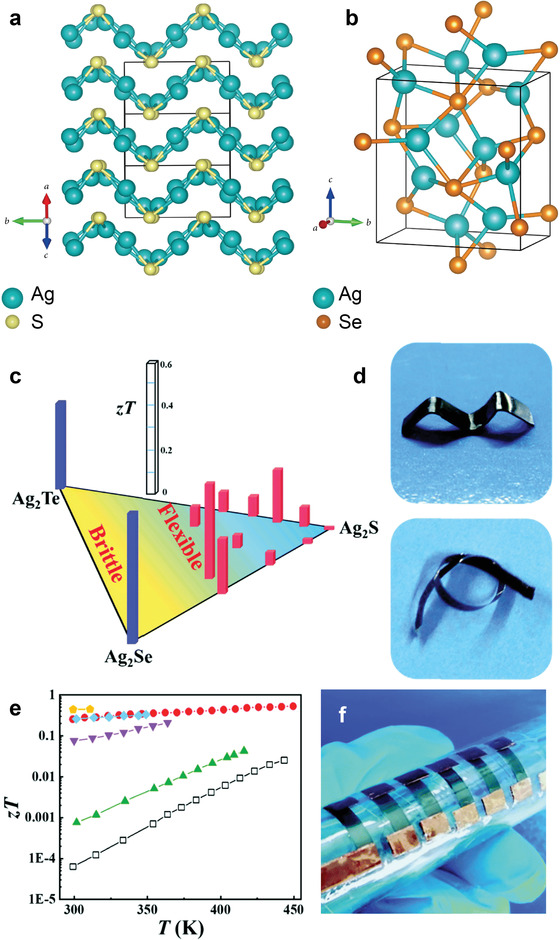
Crystal structures for a) monoclinic Ag_2_S and b) orthorhombic Ag_2_Se. Reproduced under Creative Common CC BY 4.0 license.^[^ ^154^ ^]^ Copyright 2021, The Authors. Published by AAAS. c) Flexibility‐*zT* phase diagram for Ag_2_S‐Ag_2_Se‐Ag_2_Te system. d) Twisted Ag_2_S_0.5_Se_0.5_ and Ag_2_S_0.8_Te_0.2_ samples in various shapes. e) Temperature dependence of *zT* for Ag_2_S_1−_ *_x_* Se*_x_* (*x* = 0/white, 0.1/green, 0.3/purple, and 0.5/cyan), Ag_2_S_0.8_Te_0.2_ (red), and Ag_2_S_0.5_Se_0.45_Te_0.05_ (yellow). f) Optical image of a six‐couple flexible Ag_2_S_0.5_Se_0.5_/Pt‐Rh thermoelectric device. Reproduced with permission.^[^ ^20^ ^]^ Copyright 2019, Royal Society of Chemistry.

A high degree of ductility is commonly observed in metals and metal alloys, in contrast with semiconductors and ceramic materials. Owing to its wrinkled layer structure and weak Ag—S bonds, monoclinic Ag_2_S exhibits unexpected ductility at room temperature (**Figure** **4**a–d).^[^ ^20^, ^153^, ^154^ ^]^ A study of the chemical bonding by Shi and co‐workers found that silver diffusion results in the irregular distribution of Ag—Ag and Ag—S bonds, which suppresses the cleavage of Ag_2_S, resulting in the high mechanical performance of the semiconductor.^[^ ^153^ ^]^ Stretchable thin films of Ag_2_S have been produced according to a solution‐processed synthesis on a stretchable substrate, which were capable of maintaining structural integrity at a tensile strain of 14.9%.^[^ ^155^ ^]^ Semiconducting Ag_2_S is able to accommodate large amounts of Se (50%) or Te (20%) in ternary alloys which maintain the flexibility and ductility performance of pristine Ag_2_S.^[^ ^154^ ^]^ The plastic deformations of monoclinic Ag_2_S are accessible at room temperature; this enables the usage of Ag_2_S in the creation of flexible and wearable thermoelectric devices (e.g., biosensors, smart watches and e‐skins).^[^ ^156^, ^157^, ^158^, ^159^, ^160^ ^]^

### 3.2 Silver Selenide (Ag_2_Se)

Ag_2_Se crystallizes in an orthorhombic structure (*β*‐Ag_2_Se) under ambient conditions and undergoes a polymorphic phase transition to a cubic structure (*α*‐Ag_2_Se) at an elevated temperature (409 K, 1 atm), which is stable until melting at 1170 K.^[^ ^161^ ^]^ The *β*‐Ag_2_Se phase exhibits semiconducting properties due a narrow bandgap (0.15 eV), while the *α*‐Ag_2_Se phase displays metallic superionic properties, in which Ag^+^ ions become mobile within a rigid sublattice of Se^2−^.^[^ ^162^ ^]^ Modulation of the electric transport properties of Ag_2_Se near the phase transition boundary between the orthorhombic and cubic phases has provided a means to optimize thermoelectric properties.^[^ ^45^ ^]^ Ferhat and Nagao fabricated polycrystalline orthorhombic Ag_2_Se ingots through the direct reaction of Ag and Se at 1273 K in an evacuated quartz tube, accomplishing a very high power factor of ≈3500 µW m^−1^ K^−2^ and a *zT* value of 0.96 at 300 K, which is one of the highest power factor values reported at room temperature. This high performance was ascribed to the relatively low thermal conductivity and high carrier mobility of the material.^[^ ^67^ ^]^

In recent years, numerous attempts have been made to finely tune the composition of nonstoichiometric Ag_2_Se in order to control the concentration of free carriers and optimize thermoelectric properties. This tuning typically results in an increase of average *zT* with temperature, and a decrease in electron concentration. These observations likely arise from the presence of excess of Se in the Ag_2_Se. A peak *zT* was attained at 300 K by Aliev and co‐workers, at an electron concentration of n ≈ 6.5 × 10^18^ cm^−3^.^[^ ^163^ ^]^ Lee et al. prepared Ag‐ and Se‐rich Ag_2_Se by mechanical alloying followed by pulse discharge sintering.^[^ ^37^ ^]^ It was found that excess Ag atoms or clusters increased the carrier concentration, but decreased the Hall mobility. A slight excess of Se was found to increase the Hall mobility, which had a strong effect on the increased *zT* values. Huang and co‐workers prepared bulk Ag_2_Se by melting and mechanical alloying.^[^ ^72^ ^]^ Hot‐pressing was subsequently used to obtain pellets which featured Ag‐ and Se‐rich nanoprecipitates and micropores with Se precipitation. Fine‐tuning the Ag/Se ratio to afford a slight excess of Se optimized the carrier concentration, resulting in an average *zT* of 0.83 in the nominal composition of Ag_2_Se_1.02_ over the range of 300−380 K.

Day et al. used a single parabolic band model calculation to predict a high *zT* of ≈1.0 for Ag_2_Se with a less‐than‐stoichiometric amount of Se.^[^ ^70^ ^]^ However, such high performance was not realized in their Ag‐rich samples, Ag_2+_ *_x_* Se, because the optimum carrier concentration was not reached at these compositions. Conversely, Mi et al. used Se‐rich Ag_2_Se_1+_ *_x_* to tune the carrier concentrations and electrical transport properties of Ag_2_Se.^[^ ^164^ ^]^ The introduction of a small excess of Se (Ag_2_Se_1.08_) enabled a significant reduction in the carrier concentration towards the optimum value, resulting in an improved power factor and *zT* value with a maximum of ≈1 at 401 K. Li and co‐workers demonstrated that an improvement in the power factor of polycrystalline Ag_2_Se could be achieved when a liquid‐phase sintering process was introduced.^[^ ^68^ ^]^ The greater power factor was associated with an increase in the Seebeck coefficient, resulting from an increase in the effective mass. In addition, a decreased carrier mobility was observed because of reduced electronic thermal conductivity, resulting in a *zT* value of ≈1.21 for the Ag_2_Se.

**Figure 5:**
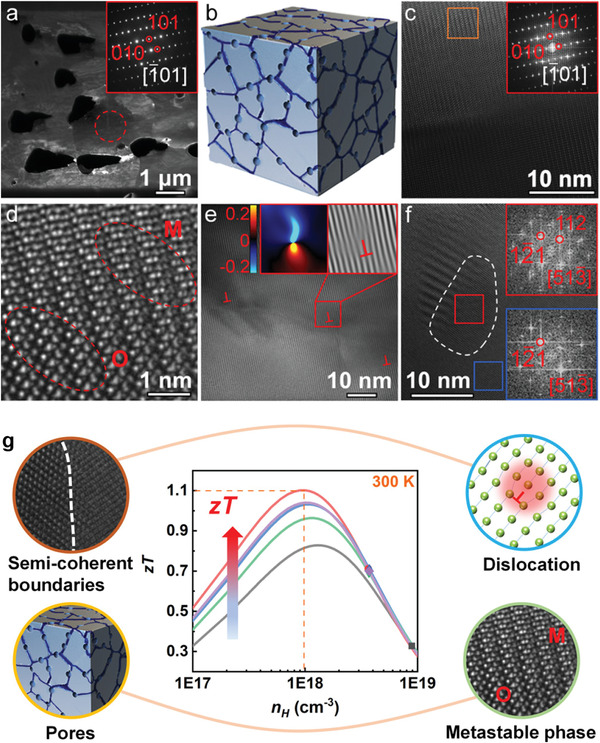
a) Dark‐field transmission electron microscopy (TEM) image of Ag_2_Se showing the grain and pore distribution. b) Schematic view for the nanopore distribution at grain interfaces. c) High‐resolution TEM image taken along the [$\bar{1}$01] zone axis and its fast Fourier transform (FFT) pattern. d) Enlarged image from the orange square area in (c) in which the “O” and “M” represent the orthorhombic phase and metastable phase of Ag_2_Se, respectively. High‐resolution TEM image showing e) high‐density dislocations and f) nanosized grains with semi‐coherent interfaces. g) Schematic illustration of Ag*_2_* Se with hierarchical pore architectures consisting of high‐density pores, a metastable phase, nanosized grains, semi‐coherent grain boundaries, high‐density dislocations, and localized strains, resulting in high thermoelectric performance at room temperature. Reproduced with permission.^[^ ^75^ ^]^ Copyright 2020, American Chemical Society.

Another effective strategy to improve thermoelectric performance is to diminish the lattice contribution to the total thermal conductivity. Chen et al. employed a method of wet mechanical alloying with spark plasma sintering to prepare hierarchically structured *β*‐Ag_2_Se.^[^ ^75^ ^]^ This structure was observed to promote strong phonon scattering and suppress lattice thermal conductivity. The low lattice thermal conductivity of ≈0.35 W m^−1^ K^−1^ at 300 K arises from the porous nature of hierarchical *β*‐Ag_2_Se structures, which also contain metastable phases, nanosized grains, semicoherent boundaries, high‐density dislocations, and localized strains. The combination of these features contributed to high *zT* values of ≈0.7 at 300 K and ≈0.9 at 390 K (**Figure** **5**). The fast fusing process of Ag_2_Se grains during sintering creates the porous structure, due to the high surface energy of nanosized Ag_2_Se powders which have been produced by a wet mechanical alloying process.^[^ ^165^ ^]^ Comparatively, conventional melting‐annealing‐sintering processes^[^ ^70^ ^]^ and manual grinding methods^[^ ^73^, ^74^ ^]^ generally yield highly densified Ag_2_Se samples. Similar porous structures have also been observed in other silver chalcogenides produced by solution methods,^[^ ^17^, ^44^, ^166^ ^]^ possibly due to the high surface energy of the nanosized powders.

A sequential manual mixing and spark plasma sintering method has been developed as a facile reaction process to synthesize silver chalcogenides from elemental powders directly.^[^ ^73^, ^74^ ^]^ In the absence of a high‐temperature annealing step, the alloying process is driven by the dissociative adsorption reaction of Se and Ag, and promoted by intermittent grinding under ambient conditions.^[^ ^74^ ^]^ In this case, a stoichiometric mixture of Ag and Se powders was reacted to form orthorhombic Ag_2_Se. Even without grinding, phase‐pure orthorhombic Ag_2_Se was obtained after standing for 13 h under ambient conditions, as a consequence of a direct reaction between solid Ag and Se vapor. The subsequent cold‐pressed stoichiometric Ag_2_Se pellet featured multiscale nanostructures, whilst hot‐pressed pellets produced by spark plasma sintering exhibited a larger average grain size, ranging from a few microns to tens of microns. In comparison with the hot‐pressed Ag_2_Se pellets, the cold‐pressed pellets exhibited lower carrier concentrations (closer to the optimal carrier concentration in Ag_2_Se), accounting for a lower electrical conductivity and a higher Seebeck coefficient. Cold‐pressed Ag_2_Se also displayed a larger weighted majority‐to‐minority carrier mobility ratio (electron/hole ratio) and lower thermal conductivity. The combination of these attributes resulted in a *zT* value of 1.2 at 390 K for cold‐pressed Ag_2_Se prepared by the solventless synthesis process at room temperature.

Wang et al. developed an aqueous solution strategy to synthesize Ag_2_E (E = S/Se/Te).^[^ ^166^ ^]^ By tuning the composition of precursors (Ag^+^/E^2−^ ratios), the thermoelectric performance of silver chalcogenides could be effectively optimized, yielding maximum *zT* values of 0.84 at 380 K for Ag_2_Se, 0.59 at 650 K for Ag_2_Te and 0.27 at 540 K for Ag_2_S. Chen et al. obtained thin polycrystalline films of Ag_2_Se from Cu_2‐_ *_x_* Se template films via a cation exchange process between Cu^+^ and Ag^+^ at room temperature (2Ag^+^ + Cu_2_Se → Ag_2_Se + 2Cu^+^).^[^ ^57^ ^]^ These polycrystalline films exhibited a maximum power factor of 825 mW m^−1^ K^−2^ and a *zT* value of 0.46 at room temperature. The Cu_2‐_ *_x_* Se films, with an average thickness of 80 nm, exhibited an electrical conductivity of ≈2.39 × 10^5^ S m^−1^ at room temperature due to the high carrier concentration of holes (Cu vacancies). This value decreased significantly to 7.5 × 10^4^ S m^−1^ following the rapid diffusion of Ag^+^ guest ions into both vacant and interstitial sites of Cu_2_Se. As a result, the value of the Seebeck coefficient switched from positive to negative, indicating the phase transformation from p‐type Cu_2_Se to n‐type Ag_2_Se.

### 3.3 Silver Telluride (Ag_2_Te)

Ag_2_Te crystallizes in a monoclinic structure (*β*‐Ag_2_Te) under ambient conditions,^[^ ^167^ ^]^ and undergoes a polymorphic phase transition to a cubic structure (*α*‐Ag_2_Te) at 423 K.^[^ ^167^ ^]^ The *β*‐Ag_2_Te phase exhibits semiconducting behavior, having a narrow bandgap of 0.06 eV, while *α*‐Ag_2_Te displays metallic superionic properties, as Ag^+^ cations are able to easily move through the cubic sublattice of Te^2−^ at high temperatures.^[^ ^161^ ^]^ The transition between these phases modifies the electrical transport properties and lattice volume of the material, which are closely related to its thermoelectric properties.

The presence of small molecules such as hydrazine, 1,2‐ethanedithiol, and ethylenediamine, on the surface of Ag_2_Te nanocrystal thin films has been demonstrated to alter the electrical transport properties of such films.^[^ ^76^ ^]^ These effects vary with the strength of the binding affinity of the molecule to the surface, which derives from the number and type of functional groups present in the molecule. The small molecules serve to scatter phonons, thus reducing thermal conductivity and allowing for higher thermoelectric performance at room temperature.

Apart from size control and the surface binding of small molecules, thermoelectric performance can also be tuned via morphological control. Without using a surfactant, Chang et al. performed a solvothermal synthesis of phase‐pure Ag_2_Te nanowires under different heating conditions.^[^ ^44^ ^]^ The initial reduction to form elemental silver was followed by the introduction of active Te in a dissolution step. The growth of the Ag_2_Te nuclei occurs preferentially in one dimension due to the base facet size and surface tension, and an overall minimization of surface energy. In addition, higher growth temperatures were shown to increase the surface energy of nanowires, resulting in interconnected nanowires. As the temperature was increased even further, dendrites were obtained. Upon densifying the Ag_2_Te nanowires to pellets, a maximum *zT* value of 0.9 was obtained at 630 K.

The introduction of holes in Ag_2_Te can be achieved by doping with a metal to increase carrier concentration. Besides the enhancement of electrical properties, the reduction of lattice thermal conductivity is also an effective way to substantially improve thermoelectric properties. For instance, 1.63% of additional silver in Ag_2_Te was shown to modulate the carrier density by affecting defects (e.g., completely ionized silver atoms at interstitial sites and vacancies), and creating both additional donor and acceptor levels.^[^ ^44^ ^]^ Owing to the optimized carrier concentration and strengthened phonon scattering, the Ag‐rich Ag_2_Te (Ag_2+_ *_X_* Te) sample in this work exhibited a maximum *zT* value of 1.1 at 623 K, which is higher than the *zT* of the binary Ag_2_Te equivalent (0.9 at 623 K).

## 4 Ternary Silver‐Based Chalcogenides

Ternary silver‐based chalcogenides can be created by reacting a silver precursor with two types of chalcogen precursors, or a silver precursor with another metal and a chalcogen precursor, resulting in products with the chemical compositions Ag‐E1‐E2 (E = S/Se/Te) and Ag‐M‐E (M = Si/K/Cu/Ga/In/Sn/Sb/Au/Bi, E = S/Se/Te), respectively (Table 1). By tuning their chemical stoichiometries (i.e., atomic ratio of Ag/E or Ag/M), electronic properties including bandgap energy, crystal structure, carrier concentration, and even mechanical characteristics can be altered for the enhancement of thermoelectric performance.

### 4.1 Ag‐E1‐E2 (E = S/Se/Te)

Ag‐E1‐E2 (E = S/Se/Te) compounds have been reported to exhibit superior thermoelectric performance compared to their binary counterparts, which is typically ascribed to the effective scattering of short‐wavelength phonons by the atomic defects, which are generated through alloying. As mentioned previously, regulating the semiconductor‐superionic phase transition, in conjunction with reduced grain size in solids, enables the balancing of electronic and thermal properties for optimized thermoelectric behavior. As a result, ternary silver‐based chalcogenides, such as Ag_4_SeS, also display lower thermal conductivities and higher *zT* values around the phase transition temperature.^[^ ^45^ ^]^

Jood et al. strived to enhance carrier mobility whilst simultaneously tuning carrier concentration.^[^ ^19^ ^]^ Their study successfully optimized *zT* values by firstly revealing structural transformations in bulk stoichiometric Ag_2_Se at room temperature, and secondly by stabilizing the main orthorhombic structure via the introduction of a slight excess of S or Se. The relationships between structural changes, carrier transport, and Ag/Se ratio were established and used to optimize *zT* values in Ag_2_Se. Consequently, a 40–70% enhancement in carrier mobility (2510 cm^2^ V^−1^ s^−1^ at 300 K) was achieved for Ag_2_SeE*_y_* (*y* ≤ 0.01, E = Se or S), together with a low lattice thermal conductivity (0.2–0.1 W m^−1^ K^−1^ over 300–375 K), which resulted from the optimized defect structure. As a result, a room‐temperature power factor of 3.2 mW m^−1^ K^−1^ and *zT* values of 0.9–1.0 between 300–375 K could be reached.

Recent work on ternary silver‐based chalcogenides has revealed that S substitution at the Se sites of Ag_2_Se can lower the phase transition temperature.^[^ ^78^ ^]^ Following S substitution, a high *zT* of 1.08 was obtained at 350 K for the composition Ag_2_S_0.4_Se_0.6_, which was 40 K lower than stoichiometric Ag_2_Se, indicating that Ag_2_Se‐based solid solutions have great potential as near room temperature thermoelectric materials.

Alloying with Se in Ag_2_S has been shown to lead to vast increases in electrical conductivity, e.g., a five‐orders‐of‐magnitude increase from 0.1 S m^−1^ for Ag_2_S to 3 × 10^4^ S m^−1^ for Ag_2_S_0.5_Se_0.5_ at 300 K was recorded.^[^ ^20^ ^]^ The considerably improved electrical conductivities are a consequence of the increasing carrier mobility which rises with increasing Se content in Ag_2_S. Alloying Se at S‐sites not only substantially reduces the bandgap, but also alters the shape of the conduction band minimum to yield a smaller carrier effective mass, which leads to higher carrier mobility. The alloying process also suppresses the lattice thermal conductivity by introducing point defects which scatter heat‐carrying phonons. This effect also helps to improve *zT* of the material. Upon compositional optimization, a delicate balance was reached between high carrier mobility and power factor, and a *zT* of 0.26 for Ag_2_S_0.5_Se_0.5_ at 300 K (Figure 4e) was achieved.

Apart from the ternary Ag–S–Se alloyed system, Chen et al. also prepared ternary Ag_2_Se_1−_ *_x_* Te*_x_* (*x* = 0.1−0.5) microstructures via wet‐mechanical alloying and spark plasma sintering.^[^ ^168^ ^]^ Alteration of Te content, band structure, carrier concentration, and carrier mobility of Ag_2_Se_1−_ *_x_* Te*_x_* was observed to manipulate electronical transport properties. The dislocations, nanograins, high‐density boundaries, Te substitutions, lattice distortions, and localized strain in Ag_2_Se_1−_ *_x_* Te*_x_* could strongly scatter phonons, and so a lattice thermal conductivity in the range of 0.21−0.31 W m^−1^ K^−1^ at 300 K was achieved.

Zhou et al. solvothermally synthesized size‐tunable Ag_2_Te nanoparticles by using 1‐dodecanethiol as both a surfactant, to confine the growth of particles, and a source of dopant sulfur.^[^ ^77^ ^]^ Without the controlled doping of sulfur, quantum size effect resulted in an enlarged bandgap and decreased concentration of charge carriers. However, the quantum effect was not prevalent in S‐doped Ag_2_Te nanoparticles, due to undefined boundary distances among nanoparticles after hot pressing. The higher charge concentration of the nanoparticles was directly related to the increasing sulfur content. In addition, a higher Seebeck coefficient was also observed in the nanosized S‐doped Ag_2_Te compared to microsized Ag_2_Te pellets, likely due to an energy filtering process which scatters the low energy charge carriers at rich interfaces in the pellets. A *zT* value of ≈0.62 was obtained for 15 nm S‐doped Ag_2_Te nanoparticles at 550 K, which was 32% higher than the bulk Ag_2_Te ingot and 114% higher than for ≈1 µm microparticles of undoped Ag_2_Te.

### 4.2 Ag‐M‐E (M = Si/K/Cu/Sn/Sb/Au/Bi, E = S/Se/Te)

Another group of ternary silver‐based chalcogenides, Ag‐M‐E (M = Cu/Sn/Sb/Au/Bi, E = S/Se/Te) have been extensively researched for potential improvements in thermoelectric performance. Li et al. demonstrated a one‐pot solvothermal synthesis of Ag_2_Se and Ag_1.9_Sn_0.1_Se granular structures with an average size of around 100 nm.^[^ ^81^ ^]^ The Sn doping enhanced the thermoelectric performance of Ag_2_Se, due to a reduced thermal conductivity and optimized power factor. The increase in electrical resistivity and Seebeck coefficient upon Sn doping was related to a decrease in electron concentration from 1.69 × 10^19^ cm^−3^ for Ag_2_Se to 1.38 × 10^19^ cm^−3^ for Ag_1.9_Sn_0.1_Se at room temperature. A peak *zT* of 0.7 was obtained at 317 K for Ag_2_Se, and partial substitution of Ag by Sn yielded a higher *zT* to 0.9 for Ag_1.9_Sn_0.1_Se at 300 K.

**Figure 6:**
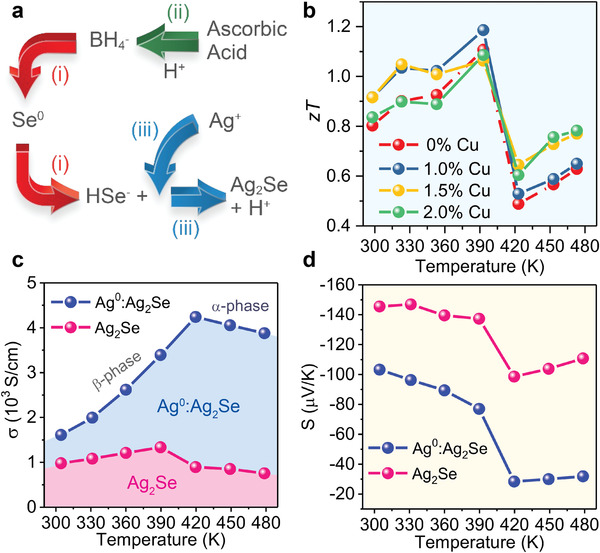
a) Schematic aqueous synthesis of Ag_2_Se at room temperature. b) Thermoelectric *zT* as a function of temperature for Ag_2_Se and *x*%Cu^+^:Ag_2_Se (*x* = 1.0, 1.5 and 2.0) samples. Temperature dependent c) electrical conductivity (*σ*) and d) Seebeck coefficient (*S*) for Ag_2_Se and Ag^0^:Ag_2_Se pellets. Reproduced with permission.^[^ ^43^ ^]^ Copyright 2022, American Chemical Society.

Recently, we have successfully synthesized binary Ag_2_Se, hybrid Ag^0^:Ag_2_Se, and ternary Cu^+^:Ag_2_Se thermoelectric materials through aqueous solution‐based approaches under ambient conditions (**Figure** **6**a). This approach enables the control of the interior composition and the components of pure and doped Ag_2_Se (as well as their phases), which can substantially influence their thermoelectric properties. In pure Ag_2_Se, *zT* values ranged from 0.8 to 1.1 between room temperature and 390 K. In comparison to pure Ag_2_Se, Cu^+^ doping improved the *zT* value to 0.9–1.2 between 300 and 393 K (Figure 6b). This increase was attributed to the enhanced electrical conductivity and the suppressed thermal conductivity which accompanies the incorporation of Cu^+^ into the lattice of Ag_2_Se at very low concentrations (*x*%Cu^+^:Ag_2_Se, *x* = 1.0, 1.5, and 2.0). On the other hand, the addition of Ag^0^ to Ag_2_Se drastically increased the electrical conductivity, leading to a more than 400% conductivity increase at 423 K. The presence of Ag^0^ in the Ag_2_Se sample not only greatly increased its electrical conductivity, but also significantly decreased its Seebeck coefficient; the synergistic combination of these effects resulted in a lower power factor than that observed for pure Ag_2_Se (Figure 6c,d).

Ternary AgCuSe nanoparticles synthesized under surfactant‐free aqueous conditions were investigated for their thermoelectric properties in the temperature range of 3 to 623 K.^[^ ^86^ ^]^ The nanoparticles exhibited strong metallic characteristics below 60 K, and exhibited n‐type semiconductor properties as the temperature was increased. This property change was accompanied by a structural transformation from a pure tetragonal phase into a mixture of tetragonal and orthorhombic phases between 60 and 480 K. After a further transition to a cubic phase above 480 K, the nanoparticles displayed p‐type semiconductor behavior. The cubic phase is comprised of a face‐centered cubic lattice of Se^2−^ with randomly distributed Ag^+^ and Cu^+^ ions amongst the tetrahedral sites, which enables the high mobility of the Ag^+^ and Cu^+^ ions and results in superionic characteristics. Accordingly, the electrical conductivity of AgCuSe dropped from 1040 to 400 S cm^−1^ as the temperature was elevated from 323 to 467 K, and further declined to 100 S cm^−1^ after the phase transition, due to the drastically increased scattering of charge carriers by highly disordered Ag^+^ and Cu^+^ ions. The Seebeck coefficient increased from −90 µV K^−1^ at room temperature to 226 µV K^−1^ at 467 K and remained nearly constant in the cubic phase. As a result, *zT* values of 0.42 at 323 K and 0.9 at 623 K were achieved and exhibited good cycling stability. The temperature‐dependent phase transition enables AgCuSe to act as either an n‐type or a p‐type thermoelectric material at different temperatures. Meanwhile, significant changes in the thermoelectric properties were observed in the presence of phase impurity‐induced compound defects at high temperatures. As a result, the *zT* of AgCuSe continuously increased to 0.6 at 450 K, whereas nonstoichiometric AgCuSe showed a considerably lower *zT* with increasing temperatures, which was attributed to the contributions from both electrons and holes.^[^ ^85^ ^]^

Likewise, AgCuS is a superionic semiconductor that undergoes temperature‐dependent p–n–p‐type conduction switching during an orthorhombic to hexagonal structural transition.^[^ ^169^ ^]^ Both the Ag vacancy concentration and Cu—S hybridized states are responsible for the p–n–p‐type conduction switching in AgCuS. Upon creating extrinsic Ag/Cu nonstoichiometry in AgCuS, the p–n–p‐type conduction switching diminishes and also results in enhanced thermoelectric properties. Particularly, the cation (Ag^+^ and Cu^+^) vacancies in AgCuS increase the p‐type carrier concentration, which in turn improve the electrical transport. Both Ag_1−_ *_x_* CuS and AgCu_1−_ *_x_* S exhibit low thermal conductivity due to the low‐energy cationic sublattice vibration caused by the movement of loosely bound Ag/Cu within the rigid anion sublattice. Ag_0.85_CuS yields a *zT* of ≈0.15, which is considerably higher than that of pure AgCuS.

Ternary Ag_3_AuSe_2_ nanoparticles were synthesized from binary Ag_2_Se nanoparticles via a colloidal approach.^[^ ^46^ ^]^ The diffusion of Au^+^ into the *β*‐Ag_2_Se lattice led to a phase transformation from orthorhombic *β*‐Ag_2_Se to a cubic Ag_3_AuSe_2_ structure with a bandgap of ≈0.2 eV.^[^ ^170^ ^]^ The densified ternary powder displayed a two‐fold decrease in electrical conductivity and 50% increase in Seebeck coefficient relative to pristine Ag_2_Se, which translated into a higher power factor. This behavior originates from charge scattering and the corresponding energy filtering of carriers with lower energy, leading to a reduction in the bipolar effect induced by the phase transition.

Ternary I–V–VI_2_ AgME_2_ compounds, such as AgBiS_2_, AgSbSe_2_, and AgBiSe_2_, are very promising thermoelectric materials, possessing enhanced ionic and electronic mobilities, and low thermal conductivities. At room temperature, bulk AgBiS_2_ crystallizes in a hexagonal phase and transforms to a cubic rock‐salt structure at ≈473 K, with disordered Ag and Bi atoms. The anharmonicity in the Bi—E bond (E = S/Se/Te) originates from the electrostatic repulsion between the stereochemically active lone pair of bismuth and the valence bonding charge of the chalcogen. The magnitude of this repulsive force is expected to increase from tellurium to sulfur due to the increase in electronegativity up the chalcogen group.^[^ ^171^, ^172^ ^]^ Biswas et al. synthesized nanocrystals of the high‐temperature rock‐salt phase of AgBiS_2_ in solution, which were kinetically stabilized at room temperature.^[^ ^90^ ^]^ The presence of a notable order‐disorder type transition in the Ag/Bi lattice, the high degree of anharmonicity in Bi—S bonds, and the nanoscale grain boundaries in AgBiS_2_ gave rise to effective phonon scattering. This resulted in a minimal lattice thermal conductivity of 0.4−0.5 W m^−1^K^−1^ between 290 and 830 K, and a *zT* value of ≈0.2 at 810 K.

**Figure 7:**
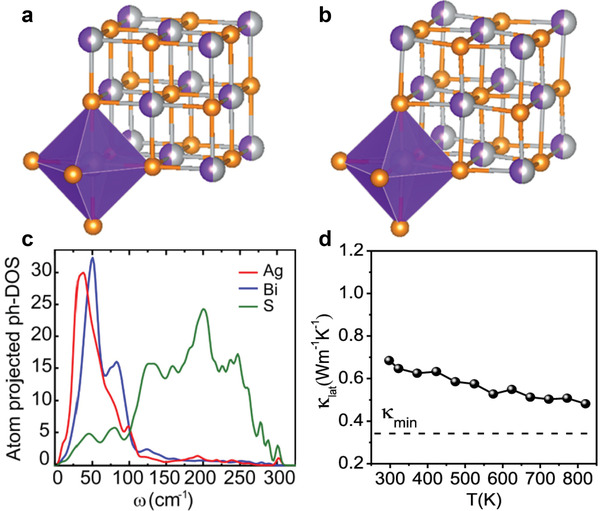
a,b) Crystal structure of AgBiS_2_ in a cubic unit cell: a) undistorted and b) small distortions of central cations away from octahedral center. The gray, violet, and orange colors represent Ag, Bi, and S atoms, respectively. c) Atom‐projected phonon density of states (PhDOS) for AgBiS_2_. d) Temperature‐dependent lattice thermal conductivity (*κ*_lat_) of cubic AgBiS_2_. The dashed line is the theoretical minimum of lattice thermal conductivity (*κ*_min_ ≈ 0.34 W m^−1^ K^−1^) of AgBiS_2_. Reproduced with permission.^[^ ^91^ ^]^ Copyright 2019, American Chemical Society.

A greater *zT* value of ≈0.7 at 820 K for AgBiS_2_ has also been reported_._^[^ ^91^ ^]^ The high *zT* performance arose from the soft lattice vibration of (predominantly) Ag and the significant lattice anharmonicity from the local structural distortions along the [011] direction (**Figure** **7**a,b), resulting from the stereochemical activity of the 6s^2^ lone pair of Bi. The soft lattice vibration of Ag and the anharmonicity from Bi also create low‐lying optical phonons which strongly scatter the heat‐carrying acoustic phonons (Figure 7c), thereby substantially suppressing the lattice thermal conductivity in cubic AgBiS_2_ close to its theoretical minimum (Figure 7d). A significant improvement in the thermoelectric performance of AgSbSe_2_ has also been observed upon the introduction of Sb deficiencies.^[^ ^83^ ^]^ In this nonstoichiometric AgSbSe_2_, an increased carrier concentration was observed without the need for additional doping, which in turn enhanced the electrical conductivity between 300 and 610 K. Additionally, AgSbSe_2_ showed low thermal conductivity as a result of the phonon scattering which arises from bond anharmonicity and a disordered cation sublattice. The superior electronic transport coupled with a low thermal conductivity led to a peak *zT* of ≈1 at 610 K for the AgSb_0.9925_Se_2_ and AgSb_0.99_Se_2_ samples.

**Figure 8:**
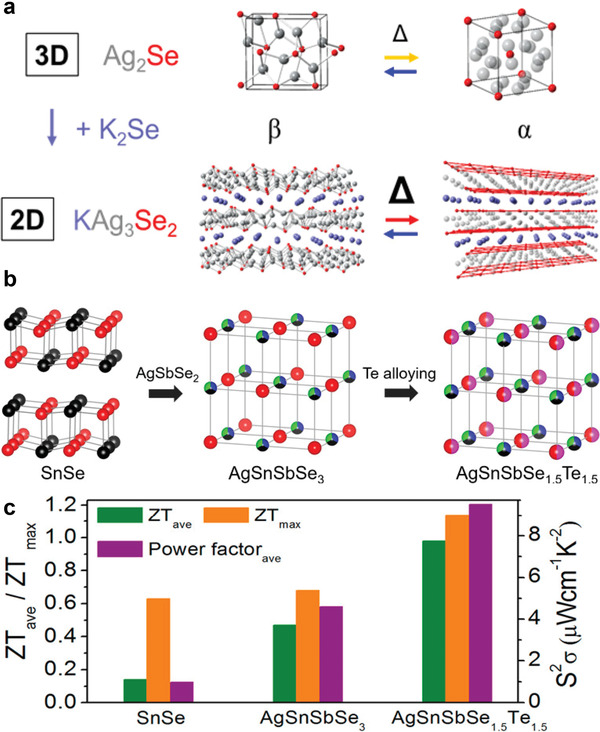
a) Crystal structures of Ag_2_Se and KAg_3_Se_2_:2D KAg_3_Se_2_ is viewed as a dimensionally reduced derivative of 3D Ag_2_Se. Reproduced with permission.^[^ ^21^ ^]^ Copyright 2018, American Chemical Society. b) Crystal structures and c) thermoelectric performance of SnSe, AgSnSbSe_3_, and AgSnSbSe_1.5_Te_1.5_. Reproduced with permission.^[^ ^116^ ^]^ Copyright 2020, American Chemical Society.

Other types of Ag‐M‐E compounds (M = K/Si, E = S/Se/Te) have also been prepared through alkali metal and nonmetal doping. As a dimensionally‐reduced 2D derivative of 3D Ag_2_Se (**Figure** **8**a),^[^ ^21^ ^]^ *β*‐KAg_3_Se_2_ was prepared by solid‐state reaction. The 2D derivative exhibited n‐type semiconductor behavior with a ≈1 eV bandgap and high electron mobility at 300 K due to a highly disperse conduction band. The monoclinic *β*‐phase transformed to a hexagonal *α*‐phase at 700 K through a first‐order phase transition, which is ≈300 K higher than the analogous transition temperature in the parent compound. An ultra‐low thermal conductivity was observed in the *β*‐phase due to the anharmonic motion of Ag ions, which impede the transport of phonons (even without extensive disordering). In this case, the dimensional reduction successfully suppressed Type I phase transitions, though desirable electronic and thermal properties were retained.

In another case, polycrystalline Ag_8_SiSe_6_ exhibited a cubic structure above 370 K with a fully disordered cation sublattice and superionic conduction properties.^[^ ^82^ ^]^ Below 370 K, the localized cations bestowed exceptional stability against the migration of Ag^+^ cations (Soret effect) under an applied temperature gradient. Electron mobilities as high as 1800 cm^2^ (V s)^−1^ were measured, with low thermal conductivity arising from Ag disorder and superstructure modulation, leading to *zT* values of 0.6 to 0.8 at 300 to 350 K, respectively.

Ternary argyrodite‐type Ag_9_GaSe_6_ is a newly recognized superior thermoelectric material due to its intrinsically low lattice thermal conductivity.^[^ ^110^, ^173^ ^]^ However, liquid‐like Ag atoms are believed to cause poor stability and performance irreproducibility. During a typical powder densification hot‐pressing process, the high pressure induces the liquid‐like Ag atoms to migrate to a position with a higher coordination number, giving rise to a metastable Ag distribution which carries a higher chemical potential and lower binding energy. In order to change such a high‐pressure‐induced high chemical potential state, an energy barrier has to be overcome, which can be realized by a subsequent annealing procedure to drive the high‐energy metastable Ag atoms back to their original crystallographic sites (of lower chemical potentials). Remarkably, hot‐pressed and annealed Ag_9_GaSe_6_ with low‐chemical‐potential Ag atoms was proven to be intrinsically stable, presenting a *zT* of ≈1.4 at 800 K after multiple measurements in Ag_8.3_Cu_0.7_GaSe_6_.^[^ ^110^ ^]^

## 5 Quaternary Silver‐Based Chalcogenides (Ag‐M1‐M2‐E)

Quaternary silver‐based chalcogenides, Ag‐M1‐M2‐E (E = S/Se/Te, M = Na/Mg/Ca/Mn/Ni/Cu/Zn/Nb/Cd/Ga/In/Sn/Sb/Ba/Pb/Bi), are made up of four different atomic constituents, which significantly increases the opportunities to find candidates with suitable band structure, composition, and crystal structure to achieve higher thermoelectric performance (Table 1). For example, cubic I‐V‐VI_2_ (where I = Cu/Ag/Au/alkali metals, V = As/Sb/Bi, VI = Se/Te) semiconductors are recognized for their intrinsically low lattice thermal conductivity (due to the strong anharmonic bonding arrangement in these compounds),^[^ ^172^, ^174^ ^]^ rendering them promising thermoelectric materials in the intermediate temperature range (500–700 K).

### 5.1 Ag_2_S + Se/Te

Alloying Ag_2_S with Se and Te can substantially improve carrier mobility, and thus increase electrical conductivity. For example, the electrical conductivity of Ag_2_S_0.5_Se_0.45_Te_0.05_ at 300 K was measured as 2.7 × 10^−4^ S m^−1^, five orders of magnitude greater than pristine Ag_2_S (0.1 S m^−1^).^[^ ^20^ ^]^ In this case, the optimized Ag_2_S_0.5_Se_0.45_Te_0.05_ composition found a balance between high carrier mobility and power factor to achieve a *zT* value of 0.44 at 300 K (Figure 4e). Importantly, the significantly enhanced *zT* values for Ag_2_(S, Se, Te) were mostly attributed to the increase in Hall carrier concentration from 1.6 × 10^14^ cm^3^ for Ag_2_S to 10^18^–10^19^ cm^3^ for Ag_2_(S, Se, Te) at 300 K. Recently, Xie et al. demonstrated that a low concentration of excess Se [Ag_2_S_0.4_(Se_0.6_Te_0.4_)_0.6_ + *y*Se (*y* = 0, 0.02, 0.03, 0.04, 0.05)] may be added to reduce the carrier concentration and enhance the thermoelectric properties further.^[^ ^96^ ^]^ A *zT* value of ≈0.3 for Ag_2_S_0.4_(Se_0.6_Te_0.4_)_0.6_ + 0.04Se at 300 K was recorded in this case, which was five times higher that of Ag_2_S_0.4_(Se_0.6_Te_0.4_)_0.6_.

### 5.2 Ag_2_S_0.7_Se_0.3_ + Cu

It is well established that the outstanding deformable and flexible thermoelectric materials (at room temperature) are Ag_2_S‐based semiconductor alloys, which exhibit n‐type conduction behavior. The n‐type semiconductor Ag_2_S_0.7_Se_0.3_ exhibits good plasticity at room temperature with a monoclinic structure analogous to room‐temperature Ag_2_S. On the contrary, its copper counterpart, Cu_2_S_0.7_Se_0.3_, adopts a hexagonal structure (analogous to the Cu_2_S medium‐temperature phase) with p‐type conduction and poor plasticity. Upon alloying, the n‐type conduction in Ag_2_S_0.7_Se_0.3_ is switched into p‐type conduction in (Ag_1−_ *_x_* Cu*_x_*)_2_S_0.7_Se_0.3_, with Cu doping when *x* is above 0.6. Above this Cu concentration, hexagonal (Ag_1−_ *_x_* Cu*_x_*)_2_S_0.7_Se_0.3_ is similar to the hexagonal Cu_2_S phase and exhibits p‐type conduction behavior. At lower Cu concentrations, the monoclinic (Ag_1−_ *_x_* Cu*_x_*)_2_S_0.7_Se_0.3_ phase is similar to monoclinic Ag_2_S, displaying n‐type conduction properties. This Cu‐ and Ag‐dependent conduction behavior is related to the dominant intrinsic defects inside the lattice, such as interstitial Ag sites,^[^ ^154^, ^175^ ^]^ and Cu vacancies.^[^ ^176^ ^]^ For instance, in monoclinic Ag_2_S, the formation energy of an interstitial Ag site is smaller than that of a Ag vacancy, implying that the interstitial sites (electron donors) are easier to generate. Conversely, the formation energy of a Cu vacancy in hexagonal Cu_2_S is smaller than that of an interstitial Cu site, suggesting that Cu vacancies (electron acceptors) are easier to generate.^[^ ^111^ ^]^

In this study by Gao and co‐workers, charged crystal defects were engineered so that both good plasticity and p‐type conduction could be simultaneously realized in (Ag_1−_ *_x_* Cu*_x_*)_2_S_0.7_Se_0.3_ (*x* = 0.7–0.8). At low Cu content (*x* = 0, 0.1, and 0.2), the good plasticity in (Ag_1−_ *_x_* Cu*_x_*)_2_S_0.7_Se_0.3_ originates from the Ag_2_S‐like crystal structure. In this structure, there is a small slippage energy (Es) and large cleavage energy (Ec) between the crystal planes, facilitating slipping without cleavage under an external stress. Unexpectedly, good plasticity was also observed in the Cu‐rich (Ag_0.2_Cu_0.8_)_2_S_0.7_Se_0.3_ samples with a hexagonal structure, as they displayed a comparable Ec/Es ratio to Ag_2_S_0.7_Se_0.3_. During slipping, the increase in the total energy of Ag‐based bonds is smaller than the Cu‐based bonds, and so the energy barrier to slipping is lower. Thus, the Ag/Cu ratio accounts for the plasticity in Ag‐containing (Ag_0.2_Cu_0.8_)_2_S_0.7_Se_0.3_ and the brittleness in Ag‐free Cu_2_S_0.7_Se_0.3_ with the same crystal structure. Gao et al. observed a maximum *zT* value of 0.42 at 800 K for p‐type (Ag_0.2_Cu_0.8_)_2_S_0.7_Se_0.3_. Introducing a small degree of Cu deficiency further enhanced the electrical conductivity and power factor, leading to significant *zT* enhancement. Crucially, a high degree of plasticity was maintained. For (Ag_0.2_Cu_0.785_)_2_S_0.7_Se_0.3_, the maximum *zT* was 0.95 at 800 K, which was two times higher than that of (Ag_0.2_Cu_0.8_)_2_S_0.7_Se_0.3_.^[^ ^111^ ^]^

### 5.3 AgCuTe + Se

Ternary AgCuTe crystallizes in a complex hexagonal structure at room temperature and undergoes a structural phase transition at about 460 K to a rock‐salt phase, in which the superionic Ag/Cu ions are disordered throughout the face‐centered cubic sublattice of Te^2−^. Stoichiometric AgCuTe is unstable at room temperature, and typically encompasses a trace amount of *α*‐Ag_2_Te as a secondary phase, suggesting the matrix to be Ag‐deficient. The intrinsic cation vacancies cause AgCuTe to be a degenerate semiconductor with a high density of hole carriers, giving rise to p‐type conduction behavior. The thermoelectric properties of AgCuTe can be improved by reducing the concentration of Ag vacancies, which is achieved by replacing Te with Se.^[^ ^88^, ^95^ ^]^ The relatively stronger Ag—Se/Cu—Se bonds compared to Ag—Te/Cu—Te bonds reduce the cationic vacancies present in AgCuTe_1−_ *_x_* Se*_x_*, lowering the hole concentrations.^[^ ^177^, ^178^ ^]^ With 10 mol% Se alloying in AgCuTe, Ag vacancies were effectively suppressed and the solubility of *α*‐Ag_2_Te in the AgCuTe matrix was enhanced.

The dynamic disorder of Ag/Cu cations in a rock‐salt phase accounts for reduced phonon frequencies and shortened mean free paths at elevated temperatures. Interestingly, hexagonal AgCuTe also exhibits low lattice thermal conductivity near room temperature (despite its localized cations), suggesting the important role of intrinsic factors, such as structural complexity and the weak bonding characteristics of cations, in suppressing lattice thermal conductivity. Based on a first principles theoretical analysis, it was found that the soft vibrations of Ag and low‐lying soft phonons both scatter heat‐carrying acoustic phonon modes, thereby reducing the lattice thermal conductivity of the hexagonal AgCuTe phase.^[^ ^88^ ^]^ Consequently, p‐type AgCuTe_0.9_Se_0.1_ reached a *zT* value of 1.6 at 700 K compared to 1.45 for pristine AgCuTe at the same temperature.

Jiang et al. compared the thermoelectric properties of AgCuTe and AgCuTe_0.9_Se_0.1_.^[^ ^95^ ^]^ It was observed that selenium alloying increased the power factor by 500%, and *zT* was improved by up to 20 times for the face‐centered cubic phase of AgCuTe_0.9_Se_0.1_, relative to the complex noncubic phase of AgCuTe. The electronic properties of the Se‐alloyed cubic phase were significantly improved, and the electrical behavior of the material switched from metallic to semiconducting. The thermal expansion of the material with decreasing temperature led to a gradual diminution of interstitial space, which made the face‐centered cubic phase less stable before it eventually transformed into a stable low‐symmetry phase. Once optimized, the Se‐alloyed (AgCu)_0.995_Te_0.9_Se_0.1_ phase exhibited a measurable *zT* of 1.1 at 350 K.

### 5.4 AgSbSe_2_ + M2 (M2 = Na/Mg/Ca/Ba/Sn/Pb/Bi)

The p‐type semiconductor AgSbSe_2_, with a cubic rock‐salt crystal structure, features disordered Ag/Sb positions and strong anharmonicity amongst the Sb—Se bonds, deriving from the Sb 5s^2^ lone pair electrons. The intrinsically low concentration of holes in pristine AgSbSe_2_ can be increased and optimized by doping with 2–4 mol% Pb^2+^ or Bi^3+^ via a solid‐state reaction, resulting in enhanced electrical transport and a significant increase in power factor.^[^ ^99^ ^]^ Due to the synergy of optimized electrical transport and low thermal conductivity, *zT* values of ≈1 and ≈1.15 at 680 K were recorded for AgSb_0.96_Pb_0.04_Se_2_ and AgSb_0.98_Bi_0.02_Se_2_, which were enhanced by 150% and 190% compared to that of pristine AgSbSe_2_, respectively.

Similarly, the hole concentration of pristine AgSbSe_2_ can also be increased by doping with Ca^2+^, which has two electrons in its valence shell and thus tends to increase the hole concentration when substitution takes place in the sublattice of Sb^3+^. Moreover, the ionic radius of Ca^2+^ dopant ions (≈99 pm) is comparable to that of the Sb^3+^ host ions (≈92 pm), likely resulting in a larger solid solubility and higher hole concentration. A *zT* value of 1.2 at 673 K was obtained for nanostructured AgSb_0.98_Ca_0.02_Se_2_, due to the increased carrier concentration and the strengthened point‐defect scattering of phonons.^[^ ^100^ ^]^

Other doping studies have explored the substitution of Sb^3+^ with monovalent Na^+^,^[^ ^98^ ^]^ divalent Mg^2+^,^[^ ^103^ ^]^ Ba^2+^,^[^ ^103^ ^]^ Mn^2+^,^[^ ^101^ ^]^ Sn^2+^,^[^ ^102^ ^]^ and trivalent Bi^3+^.^[^ ^104^ ^]^ For example, the substitution of Na^+^ in the sublattice of Sb^3+^ not only improved the carrier concentration (increasing the power factor), but also suppressed the thermal conductivity. Phonon scattering across multiple length scales was attributed to the existence of point defects, nanoscale stacking faults, and Na‐rich precipitates. As a result of these factors, a *zT* value of 0.92 at 673 K was achieved for AgNa_0.01_Sb_0.99_Se_2_.^[^ ^98^ ^]^ A *zT* value of ≈1.05 at 673 K for Mn^2+^‐doped AgSbSe_2_ was obtained, and an average *zT* value of ≈0.63 from 300 to 673 K has been attained for AgSb_0.96_Mn_0.04_Se_2_.^[^ ^101^ ^]^ In the case of AgSb_0.99_Sn_0.01_Se_2_, a *zT* value of 1.21 at 660 K was recorded, 363% higher than that of undoped AgSbSe_2_.^[^ ^102^ ^]^

### 5.5 AgBiSe_2_ + M2 (M2 = Nb/In) or X (X = Cl^−^/Br^−^/I^−^)

Similar to those materials mentioned in the previous section, n‐type I‐V‐VI_2_ AgBiSe_2_ features intrinsically low thermal conductivity due to the high degree of anharmonicity in the Bi—Se bond, and effective phonon scattering by the disordered Ag/Bi lattice. Recent efforts have been made to improve the electrical properties of the AgBiSe_2_ system, mostly focusing on elemental doping. For example, niobium doping enhanced the thermoelectric performance of AgBiSe_2_ by increasing the carrier concentration, which significantly boosted the *zT* value at 773 K from 0.5 for undoped AgBiSe_2_, to 1.0 for Ag_0.96_Nb_0.04_BiSe_2_.^[^ ^107^ ^]^ In addition, indium doping at Ag sites has been shown to more than double thermoelectric performance, with a *zT* value of 0.7 at 773 K measured for Ag_0.985_In_0.015_BiSe_2_ compared to 0.3 for pristine AgBiSe_2_.^[^ ^108^ ^]^ The thermoelectric enhancement is primarily attributed to the increased carrier concentration and the suppressed lattice thermal conductivity, which arises from the introduction of point defects as well as the increased anharmonicity of the chemical bonds due to In 5s^2^ lone pair electrons.

When AgBiSe_2_ was simultaneously doped with indium and hybridized with AgBiS_2_ through a mechanical alloying process, a peak *zT* of 0.9 was obtained at 773 K.^[^ ^179^ ^]^ The introduction of the dopant tuned the carrier concentration, while hybridization with AgBiS_2_ significantly suppressed thermal conductivity as a result of phase boundary scattering. Moreover, the mechanical alloying process further reduced the lattice thermal conductivity, because of grain size reduction and enhanced alloy scattering from the S–Se substitution. Eventually, the ball milled 80% Ag_0.99_BiSe_2_In_0.01_+ 20% AgBiS_2_ exhibited more than two times the *zT* value of pristine AgBiSe_2_.

The thermoelectric performance of AgSbSe_2_ can be also regulated by aliovalent doping. For example, the substitution of halide ions (Cl^−^/Br^−^/I^−^) into the Se^2−^ sublattice was explored to significantly increase the n‐type carrier concentration in AgBiSe_2_, giving rise to improved temperature‐dependent electronic transport properties.^[^ ^97^ ^]^ A peak *zT* value of 0.9 at 805 K was obtained for n‐type AgBiSe_1.98_Cl_0.2_, due to an increased power factor and intrinsically low thermal conductivity.

## 6 Multinary Silver‐Based Chalcogenides

Quinary silver‐based chalcogenides, Ag‐M1‐M2‐E1‐E2 (E = S/Se/Te, M = Cu/Ge/Sn/Sb/Pb/Bi), are made up of five different atomic constituents, which generally exist in a solid solution containing different principle elements with individual concentrations in a molar ratio between 5 and 35, resulting in high configurational mixing entropy (Table 1).^[^ ^180^ ^]^ The so‐called high‐entropy alloys have gained much attention in the field of thermoelectric materials owing to their increased phonon scattering, which arises from cation disorder and distorted lattices.^[^ ^181^ ^]^

An equimolar mixture of AgSbSe_2_ and SnSe forms a stable, cation‐disordered cubic rock‐salt p‐type AgSnSbSe_3_ phase, in which Ag, Sn, and Sb cations are randomly distributed over the Na sites in the NaCl lattice (Figure 8b). AgSnSbSe_3_ was found to exhibit a low lattice thermal conductivity of ≈0.47 W m^−1^ K^−1^ at 673 K, due to the combined effects of cation disorder, phonon anharmonicity, low phonon velocity, and low‐frequency optical modes.^[^ ^116^ ^]^ To further improve the thermoelectric performance of AgSnSbSe_3_, a quinary NaCl‐type solid solution, AgSnSbSe_1.5_Te_1.5_, was formed using high‐entropy engineering methods. Te alloying on the Se sites introduced randomly disordered cations and anions, which simultaneously improved the electrical and thermal transport properties of AgSnSbSe_3_, as a result of strengthened phonon scattering. This heightened scattering arises from extra point defects and lattice dislocations, as well as a higher hole carrier concentration. As a consequence of these multiple effects, the highest *zT* value of 1.14 at 723 K and average *zT* of ≈1.0 (400−773 K) were achieved in AgSnSbSe_1.5_Te_1.5_, a high‐entropy alloy (Figure 8c).

Similarly, Ag, Pb, and Bi cations in cubic rock‐salt n‐type AgPbBiSe_3_ remain statistically disordered in the Wyckoff position 4a, while the Wyckoff site 4b is occupied by Se anions. It was observed that AgPbBiSe_3_ exhibited a low intrinsic lattice thermal conductivity of ≈0.5 to 0.4 W m^−1^ K^−1^ in the temperature range 290–823 K.^[^ ^109^ ^]^ Investigations into the phonon‐transport processes of AgPbBiSe_3_ revealed a high degree of anharmonicity arising from the chemical disorder of cations. AgPbBiSe_3_ possesses bonding inhomogeneity, wherein Ag atoms are weakly bonded compared to Pb and Bi atoms in the lattice. The presence of 6s^2^ lone pairs in Pb and Bi induces strong variation in the chemical bonding of cations, and hence the lattice anharmonicity. Therefore, the fundamental origin of the phonon scattering process is the bonding heterogeneity and lattice anharmonicity arising from the 6s^2^ lone pairs of Bi and Pb. Further improvement of the thermoelectric properties was achieved by aliovalent halide doping, which increased the scattering of point defects, leading to a further reduction in the lattice thermal conductivity of AgPbBiSe_3_ to 0.23 W m^−1^ K^−1^ at 823 K. As a result, a doubling in *zT* value from 0.43 for pristine AgPbBiSe_3_ to 0.8 for AgPbBiSe_2.97_I_0.03_ at 818 K was observed.

Recently, Ag‐doped GeTe together with Sb and Pb, simultaneously formed quinary Ge_0.62_Ag_0.11_Sb_0.13_Pb_0.12_Te, and exhibited a high *zT* value of ≈2.4 at 750 K, which is significantly greater than the value of ≈1.2 obtained for binary GeTe.^[^ ^120^ ^]^ This research demonstrated that delocalized electrons resulting from increased crystal symmetry, and localized phonons from entropy‐induced disorder could coexist in high‐entropy GeTe‐based materials after the alloying of Ag, Sb, and Pb. This provides the possibility of simultaneously optimizing the electrical and thermal transport properties of the material. In comparison, ternary Ge_0.89_Ag_0.11_Te and quaternary Ge_0.77_Ag_0.11_Pb_0.12_Te only showed very low *zT* values of ≈0.4, due to the presence of impurities such as PbTe and Ag_2_Te. When impurities such as Ag_2_Te and Sb_2_Te_3_ were not present, Ge_0.74_Ag_0.11_Sb_0.13_Te displayed a greatly improved *zT* value of ≈1.6 at 750 K. Upon further alloying with a trace amount of Bi, senary Ge_0.61_Ag_0.11_Sb_0.13_Pb_0.12_Bi_0.01_Te exhibited an even greater *zT* value of ≈2.7.^[^ ^120^ ^]^ When the number of the metal elements incorporated was increased to five or more by introducing Mn, Sn, or Cd (e.g., septenary Ge_0.56_Ag_0.11_Sb_0.13_Pb_0.12_Bi_0.01_Mn_0.05_Te with a *zT* close to ≈2.7), the second phases were eliminated in all of the samples, indicating that the stabilization phenomenon of the single‐phase structure was dominated by increasing entropy.

Zhang et al. demonstrated that entropy engineering could be exploited to simultaneously yield both good thermoelectric performance and robust mechanical properties in quinary alloys (Ag*_y_* Cu_2−_ *_y_* Te_1−2_ *_x_* S*_x_* Se*_x_*).^[^ ^119^ ^]^ The coalloying of S/Se/Ag in Cu_2_Te simultaneously stabilizes a high‐symmetry hexagonal structure, extends the solubility limit of Ag, and reduces the phase transition temperature on account of increased configurational entropy. As a result, the carrier concentration is largely decreased while the effective mass is improved, contributing to a higher Seebeck coefficient and power factor. Meanwhile, the thermal conductivity is suppressed by one order of magnitude (measured as 0.29 W m^−1^ K^−1^ at room temperature), which predominantly arises from strong phonon scattering induced by lattice disorder. Notably, Cu_2_Te_0.6_S_0.2_Se_0.2_ showed a maximum *zT* value of 1.4 at 1000 K, which represents a 250% increase relative to that of pristine Cu_2_Te (*zT* = 0.4). Upon introducing Ag to Cu_2_Te_0.6_S_0.2_Se_0.2_, the *zT* value was further enhanced, reaching an average *zT* of 0.74 within 300 to 1000 K for Ag_0.1_Cu_1.9_Te_0.6_S_0.2_Se_0.2_. This represented an improvement of 470% over that of pristine Cu_2_Te (*zT* = 0.4). More importantly, Ag alloying not only improved thermoelectric properties, but also contributed to the superior mechanical properties. The incorporation of Ag significantly reinforces the compressive strength and even induces a prominent plastic deformation in Ag_0.1_Cu_1.9_Te_0.6_S_0.2_Se_0.2_, while the Ag‐free alloys exhibit brittle fracture features. The enhancement of mechanical properties can be mainly ascribed to the severe lattice‐distortion effect induced by high entropy alloying, which impedes dislocation movement and results in prominent solid solution strengthening.

Since the discovery of metal‐like Ag_2_S‐based materials, the design and exploitation of ductile thermoelectric semiconductors for high‐performance flexible devices has received much attention.^[^ ^153^, ^182^ ^]^ Semiconducting n‐type Ag_2_(Se,Te,S)‐based materials with both high thermoelectric performance and inherent ductility at room temperature have been developed, but their p‐type counterparts remain uncommon. Yang et al. systematically developed a series of p‐type ductile thermoelectric materials based on quinary AgCu(Se,S,Te) pseudoternary solid solutions, through composition and structure modulation.^[^ ^118^ ^]^ Upon alloying with S, AgCuSe_0.3−_ *_x_* S*_x_* Te_0.7_ (*x* = 0.06 and 0.08) exhibited good ductility, which originates from the increased amount of multicentered and diffused Ag—S bonds. These bonds induce a preference for slip over fracture in the material.

Beyond inherent ductility, AgCuSe_0.3−_ *_x_* S*_x_* Te_0.7_ (*x* = 0.06 and 0.08) demonstrated p‐type conduction and good thermoelectric performance. Introducing S resulted in an improved Seebeck coefficient, although the electrical conductivity was lowered due to the decreased hole concentration. Both the carrier thermal conductivity and lattice thermal conductivity decreased upon S alloying, yielding a reduced thermal conductivity. Resultantly, a *zT* value of 0.31 at 300 K was recorded for AgCuSe_0.3−_ *_x_* SxTe_0.7_ (*x* = 0.06 and 0.08). This value could be further increased to 0.45 at 300 K and 0.68 at 340 K in (AgCu)_0.998_Se_0.22_S_0.08_Te_0.7_ by inducing a small degree of cation deficiency.

## 7 Hybridized Silver‐Based Chalcogenides

The heterogeneous hybridization of silver‐based chalcogenides with other types of semiconductors has attracted increasing attention as a method to synergistically combine the favorable properties of different material domains. Thermoelectric performance may be markedly altered by inserting a secondary foreign phase into the bulk matrix of a silver‐based chalcogenide, particularly silver‐based binary, and ternary selenides (Table 1). This technique is an efficient way to scatter heat‐carrying phonons, improve Seebeck coefficients and stabilize crystal structures.

### 7.1 Hybridized Ag_2_Se

Hybridized Ag_2_Se was colloidally prepared by introducing nonmetal (Te or/and Se) or metal (Cu) nanodomains into the host matrix of Ag_2_Se,^[^ ^17^ ^]^ where an electron exchange took place at the interphase between the matrix and nanoinclusions. The n‐type nanohybrids with 5 mol% Te nanoinclusions displayed a *zT* value of 0.79 at room temperature, which accounted for nearly a twofold enhancement compared to that of pristine Ag_2_Se nanoparticles. The optimized thermoelectric performance was ascribed to electron filtering at interfaces, which improved the Seebeck coefficient without significantly compromising the electrical conductivity. In comparison, a similar loading of Cu nanoinclusions caused the opposite effect, increasing electrical conductivities and lowering Seebeck coefficients. When bulk Ag_2_Se was mixed with 5 mol% nano‐grained Cu_2_Se powders, the resulting Cu_2_Se/Ag_2_Se hybrids possessed a *zT* value of ≈0.45 at 875 K, compared to ≈0.25 at 300 K for bulk Ag_2_Se.^[^ ^54^ ^]^ The thermal stability of Ag_2_Se was greatly improved at high temperature after the incorporation of the Cu_2_Se nanoinclusions. A positive trend in the electrical conductivity was observed with increasing the amount of nanoinclusions, due to the resulting increase in carrier density. In spite of the larger electrical conductivity, the thermal conductivity was reduced in the presence of nano‐Cu_2_Se inclusions.

### 7.2 Hybridized AgInSe_2_

Among ternary chalcogenide semiconductors, I‐III‐VI_2_ AgInSe_2_ is considered a potential material for thermoelectric applications due to its low lattice thermal conductivity, which originates from Ag—Se “cluster vibrations” at low phonon frequencies.^[^ ^84^ ^]^ However, the thermoelectric application of n‐type AgInSe_2_ is limited by a bandgap of ≈1.24 eV,^[^ ^183^ ^]^ and a low carrier concentration of ≈1.3 × 10^11^ cm^−3^ at 300 K.^[^ ^84^ ^]^ In order to increase carrier concentration of AgInSe_2_, Qiu et al. introduced off‐stoichiometry by adding excess amounts of Ag to AgInSe_2_, forming Ag_1+_ *_x_* InSe_2_.^[^ ^84^ ^]^ The presence of excess Ag created interstitial Ag atoms, thereby increasing the electron density of the material. Moreover, the excess Ag (i.e., Ag_1.02_InSe_2_) increased the carrier concentration to 1.6 × 10^16^ cm^−3^, which accounted for 2–3 orders of magnitude enhancement relative to that of stoichiometric AgInSe_2_. As such, the increase in electronic transport properties, in conjunction with unchanged and low thermal conductivity, contributed to a *zT* value of 1.1 at 900 K, which was a 62% increase compared to that of the stoichiometric AgInSe_2_ counterpart.

**Figure 9:**
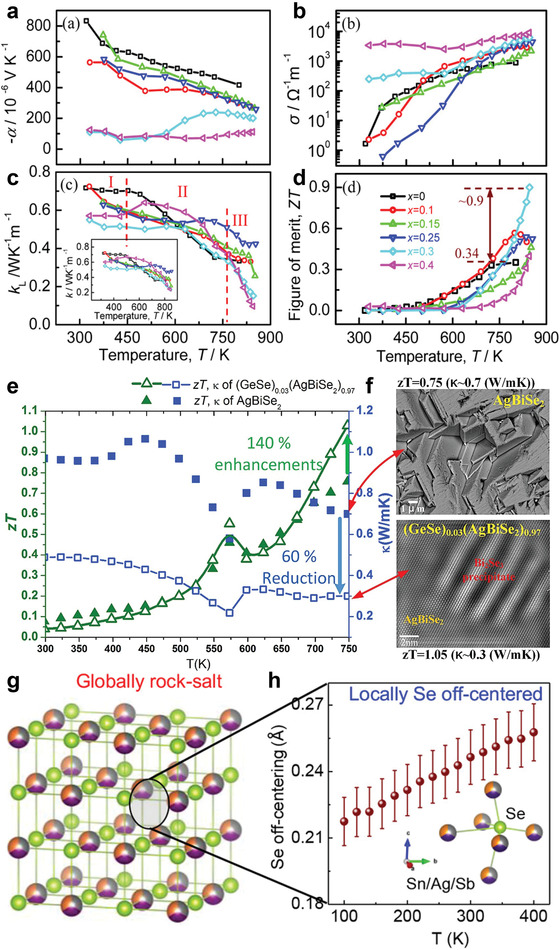
Temperature dependent a) Seebeck coefficient (*α*), b) electrical conductivity (*σ*), c) lattice thermal conductivity (*κ*_L_), d) thermoelectric *zT* of Ag_1+2_ *_x_* InSe_2+_ *_x_* (*x* = 0–0.40). An inset in (c) is the total thermal conductivity. Reproduced with permission.^[^ ^127^ ^]^ Copyright 2020, American Chemical Society. e) Temperature‐dependent *zT* and thermal conductivity of (GeSe)_0.03_(AgBiSe_2_)_0.97_ and pristine AgBiSe_2_ alloys. f) Backscattered electron image of AgBiSe_2_ and inverse fast Fourier transform (FFT) image, showing the formation of Bi_2_Se_3_ nanoprecipitate embedded in the Ge‐doped AgBiSe_2_ matrix. Reproduced with permission.^[^ ^40^ ^]^ Copyright 2017, Elsevier. g) Rock‐salt cubic structure of (SnSe)_0.5_(AgSbSe_2_)_0.5_. h) Magnitude of Se distortion along [111] direction with increasing temperature. Reproduced with permission.^[^ ^126^ ^]^ Copyright 2021, American Chemical Society.

Additionally, a trace amount of a secondary Ag_2_Se phase was found in the AgInSe_2_ compound during solid‐state reaction. As known, Ag_2_Se has a higher carrier concentration and superior electrical properties than AgInSe_2_. In order to utilize the intrinsic properties of Ag_2_Se, Zhong et al. prepared a AgInSe_2_‐based hybrid comprising a AgInSe_2_ primary phase and a Ag_2_Se secondary phase, Ag_1+2_ *_x_* InSe_2+_ *_x_* (*x* = 0−0.4).^[^ ^127^ ^]^ The in situ formation of Ag_2_Se species in the AgInSe_2_ matrix played the dual role of enhancing the carrier concentration/electrical properties, as well as promoting phonon scattering at the Ag_2_Se/AgInSe_2_ heterointerfaces (i.e., atomic scale lattice distortion and phase interfaces), thereby reducing the lattice thermal conductivity of the hybrid. Consequently, a *zT* value of ≈0.9 was reached at 846 K, a 2.7 times enhancement in thermoelectric performance compared to pristine AgInSe_2_ (**Figure** **9**a–d).

### 7.3 Hybridized AgInTe_2_

Banik et al. demonstrated that co‐doping of In and Ag in SnTe–AgInTe_2_ (i.e., Ag*_x_* In*_x_* SnTe_1+2_ *_x_*) leads to an improved thermoelectric performance over a broad temperature range (300−860 K).^[^ ^115^ ^]^ The co‐dopants (In and Ag) play distinct and complementary roles in tuning the valence band structure of SnTe. Particularly, In doping creates resonance levels inside the valence bands, thereby improving the Seebeck coefficient at room temperature. On the other hand, the incorporation of Ag leads to an increase in the principal bandgap of SnTe, thus causing a decrease in the energy separation between two valence bands (light‐ and heavy‐holes valence bands), also resulting in an improved Seebeck coefficient. Additionally, Ag doping boosts the p‐type carrier mobility of SnTe. The synergistic effect of resonance level formation and the convergence of valence bands significantly improves the electronic transport properties of Ag*_x_* In*_x_* SnTe_1+2_ *_x_*, leading to a maximum *zT* of 1 at 856 K for Ag_0.025_In_0.025_SnTe_1.05_, which is substantially higher than that of undoped SnTe.

### 7.4 Hybridized AgBiSe_2_

Ternary I‐V‐VI_2_ AgBiSe_2_ crystallizes in a hexagonal phase at room temperature, before undergoing structural phase transitions to a rhombohedral phase at 523 K and a cubic phase at 723 K. The abrupt changes in lattice constants and crystal structures during phase transitions can be stabilized between 300 and 800 K by alloying with PbSe in an entropy engineering process.^[^ ^128^ ^]^ The resultant (PbSe)*_x_* (AgBiSe_2_)_1−_ *_x_* (*x* = 0.3) solid solutions with 1% Br possessed unique locally distorted cubic lattices that yielded low lattice thermal conductivities, approaching the glass limit between 300 and 800 K. Collectively, a peak *zT* value of 0.8 at 800 K and an average *zT* value of 0.42 were obtained for cubic n‐type (PbSe)_0.3_(AgBiSe_2_)_0.7_ solid solutions doped with 1% Br.

When AgBiSe_2_ was doped with Ge, the resultant (GeSe)_0.03_(AgBiSe_2_)_0.97_ compound exhibited a low thermal conductivity of 0.3 W m^−1^ K^−1^, due to the formation of faceted rhombohedral Bi_2_Se_3_ (20–40 nm) embedded within the AgBiSe_2_ matrix.^[^ ^40^ ^]^ The combined effects of Bi_2_Se_3_ nanoprecipitates and mass fluctuations/superlattice enhance phonon scattering and suppress thermal conductivity, resulting in a *zT* value of 1.05 for (GeSe)_0.03_(AgBiSe_2_)_0.97_, which was 140% higher than that of pristine AgBiSe_2_ (Figure 9e,f).

AgBiSe_2_ can be introduced as an alloying material to tune the electronic structure and thermoelectric properties of orthorhombic and cubic SnSe.^[^ ^124^ ^]^ Upon AgBiSe_2_ alloying, the layered orthorhombic phase is stabilized in (AgBiSe_2_)*_x_* (SnSe)_1‐_ *_x_* (0 ≤ *x* ≤ 0.28), which is typical for narrow bandgap semiconductors. With a further increase in the concentration of AgBiSe_2_ (0.3 ≤ *x* ≤ 0.7), the bandgap closes and the high‐pressure cubic rock‐salt phase of SnSe is effectively stabilized at ambient temperature and pressure. The stabilization of the cubic structure arises from the increase in configurational entropy due to a higher atomic disorderliness in the system, resulting from the solid solution mixing of AgBiSe_2_ and SnSe. Interestingly, pure cubic SnSe displayed a topological crystalline insulator phase, whereas cubic (AgBiSe_2_)*_x_* (SnSe)_1‐_ *_x_* (*x* = 0.33) exhibited a semi‐metallic electronic structure with overlapping conduction and valence bands. Additionally, the cubic polycrystalline (AgBiSe_2_)*_x_* (SnSe)_1‐_ *_x_* (*x* = 0.30) showed n‐type conduction at room temperature, while orthorhombic (AgBiSe_2_)*_x_* (SnSe)_1‐_ *_x_* (0 ≤ *x* ≤ 0.28) retained its p‐type character. Notably, the p‐type polycrystalline orthorhombic (AgBiSe_2_)_0.22_(SnSe)_0.78_ yielded a *zT* value of 1.3 at 823 K due to the crystal and electric structural optimizations.

### 7.5 Hybridized AgSbSe_2_

The interdependent thermoelectric parameters of p‐type AgSbSe_2_ can be simultaneously improved by combining second‐phase nanostructuring and carrier engineering approaches. Using these approaches, in combination with the intrinsically strong Sb—Se bond anharmonicity, a *zT* value of ≈1.1 at 635 K for 2 mol% ZnSe/AgSbSe_2_ was recorded, which is 185% higher than that of pristine AgSbSe_2_.^[^ ^125^ ^]^ In this instance, the concentration of ZnSe in AgSbSe_2_ was varied, causing a transition from a solid solution system to a phase‐separated structure, and significantly affecting the electronic and thermal transport properties. At a low concentration of <2 mol% ZnSe, a solid solution of AgSbSe_2_/ZnSe was obtained, while a phase separation occurred between 2 and 8 mol% ZnSe, due to the formation of ZnSe nanostructures with different sizes and interfaces in the AgSbSe_2_ matrix. A concentration of 2 mol% ZnSe was found to be the optimal amount for p‐type doping in AgSbSe_2_, increasing the hole concentration and thus boosting the electrical transport properties. In addition, nanoscale endotaxial ZnSe precipitates were understood to act as phonon scattering centers, contributing to a reduction in the lattice thermal conductivity. Overall, the concentration of dopants, size of precipitates, and the interfaces of precipitates in the matrix were crucial in determining the electronic transport properties. At the same time, secondary phase nanostructuring and the intrinsically strong Sb—Se bond anharmonicity (within a disordered cation sublattice) enabled effective phonon scattering, improving the thermoelectric performance of the AgSbSe_2_/ZnSe system.

Structural transformations have a significant effect on thermal transport and thermoelectric properties. Typically, transformations occur from less symmetric structures to more symmetric structures with an increase in temperature. The change from a more symmetric to a less symmetric structure upon heating is rare, and this uncommon phenomenon is known as emphanisis.^[^ ^184^ ^]^ Rock‐salt (SnSe)_0.5_(AgSbSe_2_)_0.5_ exhibits rare emphanitic behavior due to the local distortion of Se along the [111] direction (Figure 9g,h).^[^ ^126^ ^]^ Local off‐centering of Se results in a local bonding hierarchy with three short and three long M—Se bonds (M = Sn/Ag/Sb) within the average rock‐salt lattice, impeding phonon propagation and reducing lattice thermal conductivity. The presence of off‐centering and consequent local bonding heterogeneity fosters a low lattice thermal conductivity in (SnSe)_0.5_(AgSbSe_2_)_0.5_. After subsequent germanium doping to enhance electrical properties, a *zT* value of 1.05 was achieved for (SnSe)_0.5_(AgSb_1−_ *_x_* Ge*_x_* Se_2_)_0.5_ (*x* = 0.06) at 706 K.

### 7.6 Hybridized Ag_2_Se and Ag_3_AuSe_2_

**Figure 10:**
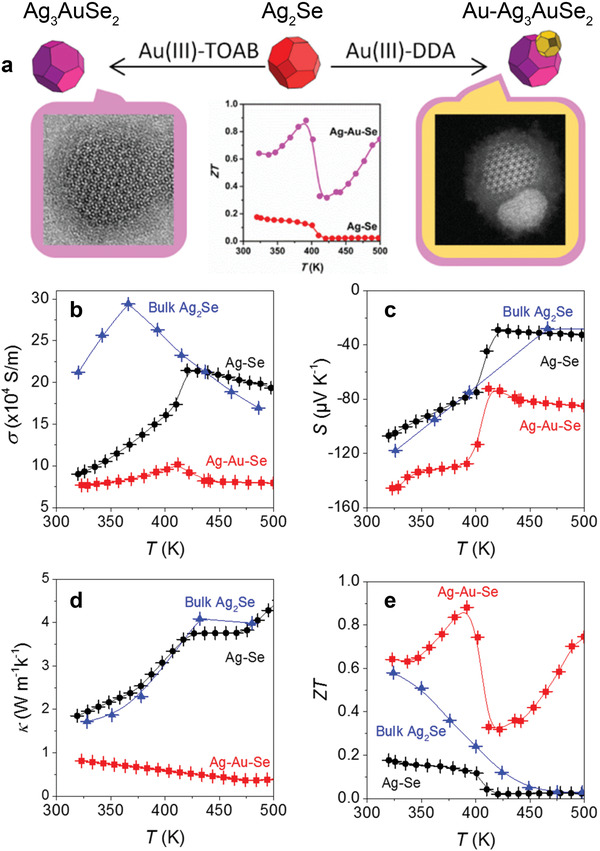
Schematic colloidal synthesis of alloyed and hybrid nanoparticles based on the Ag‐Au‐Se ternary system. Temperature dependent a) electrical conductivity (*σ*), b) Seebeck coefficient (*S*), c) thermal conductivity (*κ*), and d) thermoelectric *zT* of binary Ag_2_Se nanomaterial, Ag–Au–Se ternary nanocomposite, and Ag_2_Se ingot reference (bulk Ag_2_Se). Reproduced with permission.^[^ ^46^ ^]^ Copyright 2016, American Chemical Society.

Hybridized nanoscale Ag–Au–Se systems, such as Au–Ag_2_Se and Au–Ag_3_AuSe_2_, can be prepared from binary Ag_2_Se nanoparticles with Au via a colloidal approach.^[^ ^46^ ^]^ In this case, the presence of Au and Ag_3_AuSe_2_ resulted in a lower thermal conductivity; this decrease was due to more efficient scattering of phonons at grain boundaries, which was enhanced by the acoustic impedance mismatch of the Ag_2_Se, Au, and Ag_3_AuSe_2_ domains. As a result, the occurrence of metallic Au and Ag_3_AuSe_2_ phases within a Ag_2_Se matrix induced a sevenfold boost in thermoelectric performance, yielding a *zT* value of 0.88 at 390 K relative to 0.12 for the binary Ag—Se analog (**Figure** **10**).

## 8 Composited Silver‐Based Chalcogenides

Conventional inorganic nonmetal crystals exhibit poor flexibility due to strong ionic or covalent bonding. At low temperatures, monoclinic Ag_2_S is known to display reasonable flexibility, though it exhibits the poorest thermoelectric performance amongst the silver chalcogenides. Conversely, orthorhombic Ag_2_Se and monoclinic Ag_2_Te lack mechanical flexibility, but display far better thermoelectric performance. For instance, orthorhombic Ag_2_Se is a brittle material with a relatively small bandgap (≈0.2 eV), a high carrier mobility (≈10^3^ cm^−2^ V^−1^ S^−1^) and good electrical conductivity (≈10^5^ S m^−1^) at room temperature. Upon compositional optimization, these silver chalcogenides reached a delicate balance between high carrier mobility, power factor, *zT* value and good mechanical flexibility, presenting *zT* values of 0.26 and 0.44 for Ag_2_S_0.5_Se_0.5_ and Ag_2_S_0.5_Se_0.45_Te_0.05_ at 300 K, respectively (Figure 4c–f).^[^ ^20^ ^]^

**Figure 11:**
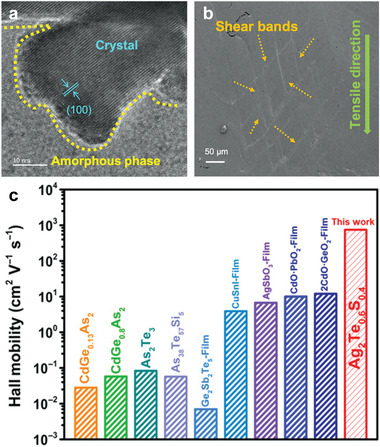
a) High resolution TEM image of Ag_2_Te_0.6_S_0.4_ showing a typical crystallite in the amorphous matrix. b) SEM image of the fracture surface showing crossing shear bands. c) Room temperature Hall mobility in comparison with other amorphous inorganic materials. Reproduced with permission.^[^ ^79^ ^]^ Copyright 2020, AAAS.

Recent work found that amorphized Ag_2_Te_1‐_ *_x_* S*_x_* possesses both good flexibility and high thermoelectric performance.^[^ ^79^ ^]^ The addition of S to Ag_2_Te led to amorphization of Ag_2_Te_1‐_ *_x_* S*_x_* due to the small size and random dispersion of crystallites (**Figure** **11**a). The unique disordered structure in Ag_2_Te_0.6_S_0.4_ gives rise to exceptional flexibility, which is mainly ascribed to the formation and evolution of shear bands which account for the plasticity of bulk metallic glasses (Figure 11b). In comparison to ductile crystalline Ag_2_S, which displays a lower carrier concentration (≈1.4 × 10^14^ cm^−3^) and poorer electrical conductivity,^[^ ^153^ ^]^ amorphized Ag_2_Te_0.6_S_0.4_ exhibits low lattice thermal conductivity due to its structural disorder, without a compromise in electrical transport properties. The Hall mobility and carrier concentration of Ag_2_Te_0.6_S_0.4_ (8.6 × 10^18^ cm^−3^) are an order of magnitude higher than other amorphous inorganic materials (Figure 11c). Accordingly, a *zT* value of 0.22 at room temperature and 0.70 at 573 K was achieved in flexible Ag_2_Te_0.6_S_0.4_ glass.

Another ductile Ag‐based thermoelectric material, Ag_20_S_7_Te_3_, has been developed for its good shape conformability.^[^ ^80^ ^]^ The outstanding shape conformability at room temperature derives from the low stacking fault energy in the (10$\bar{1}$)[010] slip system. Similarly to *α*‐Ag_2_S, the three‐point bending test revealed that Ag_20_S_7_Te_3_ has a large bending strain above 15% without cracking,^[^ ^153^ ^]^ Other semiconductors and ceramics are not able to withstand strains more than 3%.^[^ ^185^, ^186^ ^]^ The nano hardness and average Young's modulus of Ag_20_S_7_Te_3_ were determined to be 0.23 and 16.4 GPa, respectively. Both values are lower than the equivalent figures for *α*‐Ag_2_S, which displays a nano hardness of 0.44 GPa and Young's modulus of 27.1 GPa. Ag_20_S_7_Te_3_ exhibits a Vicker's hardness of 21.9 GPa, which is lower than *α*‐Ag_2_S (30.7 GPa),^[^ ^80^ ^]^ GaAs (750 GPa),^[^ ^187^ ^]^ InP (510 GPa)^[^ ^187^ ^]^ and InSb (250 GPa),^[^ ^188^ ^]^ but similar to ductile metals such as Cu (50 GPa) and Au (25 GPa).^[^ ^189^ ^]^

Apart from the shape conformability, Ag_20_S_7_Te_3_ also possesses a narrow bandgap of 0.29 eV (smaller than Ag_2_S),^[^ ^153^ ^]^ implying its potential as a thermoelectric material for near room temperature applications. The high carrier mobility and low lattice thermal conductivity observed in Ag_20_S_7_Te_3_ give rise to a maximum *zT* of 0.80 at 600 K, which is comparable with commercial Bi_2_Te_3_‐based alloys.^[^ ^190^ ^]^

Although silver‐based chalcogenides exhibit good thermoelectric performance at near room temperature, they are still generally rigid or brittle, and hence are unsuitable for assembling flexible thermoelectric devices. Flexible thermoelectric materials derived from chalcogenide/polymer composites have emerged as promising candidates for future self‐powering technology and battery‐less sensors. Chalcogenide/polymer composites can simultaneously present both a high‐power factor due to the inorganic filler, and a high flexibility and a low thermal conductivity originating from the polymer component (Table 1).

In order to increase flexibility without greatly compromising thermoelectric performance, a variety of polymers, such as poly(3,4 ethylenedioxythiophene):poly(styrenesulfonate) (PEDOT:PSS), polyaniline (PANI), polypyrrole (PPy), and polyvinylidene fluoride (PVDF), have been investigated in flexible composite films. Preparation techniques have included physical mixing, drop casting, and vacuum‐assisted filtration methods, but the thermoelectric performance is far from satisfactory so far in these instances.^[^ ^191^, ^192^, ^193^ ^]^ In addition, various other substrates, including nylon,^[^ ^47^ ^]^ cellulose,^[^ ^140^ ^]^ polyimides,^[^ ^194^ ^]^ and polyethylene naphthalates,^[^ ^141^ ^]^ have also been examined in studies concerning flexible thermoelectrics.

### 8.1 Functional Polymers

Functional polymers possess excellent flexibility and low thermal conductivity, thus they are suitable for the production of composites with silver‐based thermoelectric materials through a variety of solution processing methods or deposition techniques. A good degree of inherent flexibility enables these polymers to conform to the curvature of the host surface. A flexible Ag_2_Se nanowires/PEDOT:PSS thermoelectric composite film was fabricated via a solution mixing and drop casting method, exhibiting a power factor of 178.59 µW m^−1^ K^−2^ at 300 K (at 80 wt% Ag_2_Se nanowires).^[^ ^139^ ^]^ In another study, an electron‐conducting thin film comprised of *β*‐Ag_2_Te nanowires and poly([N,N′‐bis(2‐octyldodecyl)‐naphthalene‐1,4,5,8‐bis(dicarboximide)‐2,6‐diyl]‐alt‐5,5′‐(2,2′‐bithiophene)) yielded a peak electrical conductivity of 0.61 S cm^−1^ and a Seebeck coefficient of −126 µV K^−1^, corresponding to a power factor of ≈1 µW m^−1^ K^−2^ in the 85 wt% Ag_2_Te film at room temperature.^[^ ^146^ ^]^

#### 8.1.1 PVDF Substrates

A freestanding and flexible Ag_2_Se/PVDF composite film with a filler content of up to 90.5 wt% was prepared via the mixing of PVDF dendricolloids and Ag_2_Se nanowires, followed by filtration and cold pressing.^[^ ^137^ ^]^ The electrical conductivity of the Ag_2_Se/PVDF film increased from ≈6 to 206 S cm^−1^ as the Ag_2_Se/PVDF mass ratio was varied from 1:2 to 1:9.5. Meanwhile, a maximum Seebeck coefficient of −115 µV K^−1^ was obtained at a mass ratio of 1:5, and a peak power factor value of 189 µW m^−1^ K^−2^ was recorded at room temperature. The flexibility of the composite film was rationalized by the formation of a long‐range grapevine‐like network of soft PVDF dendritic particles which were entangled with abundant Ag_2_Se nanowires. After 1000 bending cycles, a 15.8% decrease in electrical conductivity was observed. A free‐standing Ag_2_Te/PVDF composite film was prepared by fusing 1D Ag_2_Te nanoshuttles with PVDF.^[^ ^147^ ^]^ With a mass ratio of 3.5:1, the thermoelectric films demonstrated a high electrical conductivity of 8600 S m^−1^, and a Seebeck coefficient of −60 µV K^−1^ at room temperature, resulting in a power factor of over 30 µW m^−1^ K^−2^. Further results of bending cycle tests indicated negligible performance change after 1000 bending cycles.

#### 8.1.2 Nylon Membranes

Recently, a series of *β*‐Ag_2_Se films on nylon were prepared by depositing Ag_2_Se nanowires onto a nylon membrane with vacuum‐assisted filtration, followed by a hot pressing procedure.^[^ ^47^ ^]^ The flexible films exhibited a power factor of 987 µW m^−1^ K^−2^ at 300 K and retained 93% and 80% of their original electrical conductivity after 1000 and 1500 bending cycles, respectively. The flexibility was ascribed to the combination of the flexible nylon membrane and the intertwined Ag_2_Se film, which featured numerous high‐aspect‐ratio Ag_2_Se nanograins. Nevertheless, the optimal power factor of the Ag_2_Se film is incomparable with that of bulk Ag_2_Se (3500 µW m^−1^ K^−2^).

The addition of Cu to nylon films has also been investigated. A power factor of 2231.5 mW m^−1^ K^−2^ at 300 K was recorded, which was more than double that of the Ag_2_Se film discussed above.^[^ ^132^ ^]^ The superior power factor results from the optimized carrier transport of the Ag_2_Se/Ag/AgCuSe composite film (with a molar ratio of Ag/Cu/Se = 4:1:3), and the interfacial energy filtration effect. The carrier concentration was enhanced via the injection of electrons from Ag into the conduction band of the Ag_2_Se and AgCuSe phases,^[^ ^195^ ^]^ while the energy filtering effect at the organic/inorganic heterointerface helped to maintain the magnitude of the Seebeck coefficient.

Ag_2_Se/nylon composites were also prepared by the vacuum‐assisted filtration of thick Ag_2_Se nanowires or nanosheets.^[^ ^129^ ^]^ The nanowires were synthesized at 40 °C, in order to suppress the preferential growth of Ag_2_Se grains along the (00l) direction which occurs at elevated temperatures. The change in morphology induced the formation of a compact film with ≈90% theoretical density after hot pressing. Collectively, the high density of the Ag_2_Se film and the tuned orientation of Ag_2_Se grains greatly enhanced the power factor to 1882 µW m^−1^ K^−2^ (with 90.7% retention after 1000 bending cycles) and *zT* value of ≈0.8 was measured at 300 K, which is a ≈30% increase relative to the thin Ag_2_Se nanowire film synthesized at room temperature.

**Figure 12:**
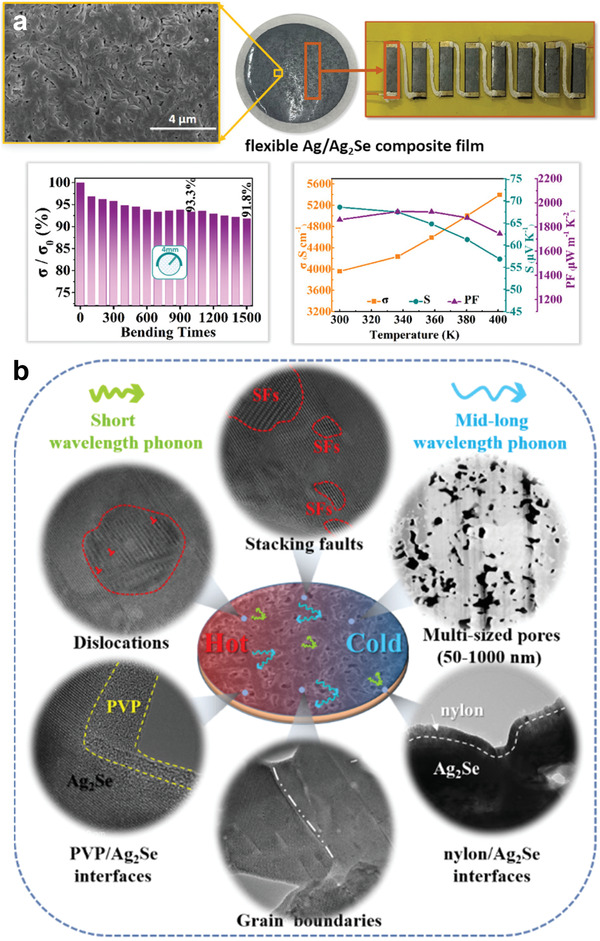
a) Thermoelectric performance of the flexible Ag/Ag_2_Se/nylon composite film and the assembled thermoelectric generator (TEG). Reproduced with permission.^[^ ^130^ ^]^ Copyright 2021, American Chemical Society. b) Schematic illustration of phonon scattering mechanisms in the PVP/Ag_2_Se composite thermoelectric film. Reproduced with permission.^[^ ^133^ ^]^ Copyright 2021, Elsevier.

Similarly, the wet chemical synthesis of Ag and Ag_2_Se nanostructures was followed by vacuum‐assisted filtration to form Ag/Ag_2_Se composite films on nylon membranes, which were then hot pressed.^[^ ^130^ ^]^ A film with a molar Ag/Se ratio of 3.5:1 exhibited a very high electrical conductivity of 3958 S cm^−1^, due to the presence of a highly conducting Ag phase. The optimized composite film achieved a power factor of 1861 µWm^−1^ K^−2^ at room temperature and retained 93% of the original electrical conductivity after bending 1000 times (**Figure** **12**a).

Ag/Ag_2_Se nanodendrites have been successfully formed using a microwave‐assisted synthesis method.^[^ ^131^ ^]^ Through tuning the molar ratios of Ag/Se, the power factor was optimized to ≈2436 µW m^−1^ K^−2^ at room temperature (with a Ag/Se molar ratio of 2.3:1). Apart from the excess Ag phase which significantly contributed to the high electrical conductivity of the composite film, the nanodendrites also have more contact points which can be welded together during the hot‐pressing process. This results in a denser film with enhanced carrier transport. In addition, Ag has a lower work function value (≈4.3 eV) compared to Ag_2_Se (≈6.79 eV), and therefore an ohmic contact could be established between the Ag and Ag_2_Se phases, which enhanced the electrical conductivity of the film. After subjection to a compressive force and a tensile force (1000 bending cycles), the Ag/Ag_2_Se composite film retained 94% and 87% of the electrical conductivity, respectively.

#### 8.1.3 Cellulose Papers

A series of Ag_2_Se nanoparticles with Ag/Se molar ratios varying from 1.9 to 2.5 were synthesized via a solvothermal reaction and cold‐pressed onto copy paper.^[^ ^140^ ^]^ The highest power factor value of 2450.9 µW m^−1^ K^−2^ at 303 K was achieved in the paper‐supported Ag_2.3_Se film after annealing in an inert atmosphere. A flexible paper‐supported thermoelectric module was then assembled by series‐connecting four pieces of annealed Ag_2.3_Se film. This device achieved an output power density of 5.80 W m^−2^ at a temperature difference of 25 K.

In a similar work, flexible n‐type Ag_2_Te nanowire films on paper substrates were constructed via a glass fiber‐assisted cold pressing technique.^[^ ^144^ ^]^ By increasing the compressive stress, a continuous and dense thin film on a paper substrate was formed. This was accompanied by the disappearance of grain boundaries in the Ag_2_Te nanowire film. As a result, the electrical conductivity of the paper‐supported Ag_2_Te nanowire film was improved, resulting in a maximum power factor of 192 µW m^−1^ K^−2^ at 468 K, while the power factor at room temperature was approximately half of this value. The film demonstrated good flexibility, with a 20% decline in power factor value after 500 bending cycles. A thermoelectric module assembled from ten legs of the paper‐supported Ag_2_Te nanowire film exhibited an open‐circuit voltage of between 11 and 60 mV as temperature was raised from 20 to 80 K.

Zeng et al. attempted to improve the room‐temperature thermoelectric performance of flexible Ag_2_Te on filter paper by welding of Ag_2_Te nanowires using vacuum filtration and drop‐coating methods.^[^ ^145^ ^]^ The welded Ag_2_Te nanowire film exhibited an electrical conductivity of 15335 S m^−1^ at room temperature, which was twice as large as the nonwelded Ag_2_Te nanowire film. This enhancement was ascribed to the interatomic bonding between the nanowires; closer physical connections increased the carrier mobility in the welded film compared to the non‐welded film. A power factor of 152 µW m^−1^ K^−2^ at room temperature was obtained for the welded Ag_2_Te nanowire film, which is nearly two times higher than Ag_2_Te–glass fiber films.^[^ ^144^ ^]^ However, the electrical resistance increased by 30% after 1000 bending cycles, likely due to microcracking on the film after numerous bends.

### 8.2 Hybrid Polymer Substrates

A series of Ag_2_Se/polymer composite films on nylon membranes were developed for their enhanced flexibility. The films were created by an in situ synthesis of polymer‐coated Ag_2_Se nanostructures, followed by vacuum‐assisted filtration of the nanostructures onto a nylon membrane and a subsequent hot‐pressing step. Polyvinylpyrrolidone (PVP) is typically used in these cases as a polymer adhesive to hinge Ag_2_Se grains.^[^ ^133^ ^]^ In this instance, it was revealed that the PVP nanolayer impeded the sintering of Ag_2_Se nanostructures, producing a porous microstructure composite film in which most Ag_2_Se grains had coherent interfaces. The PVP/Ag_2_Se composite film achieved a power factor of 1910 µW m^−1^ K^−2^ at room temperature and showed good flexibility, retaining 94.5% of the initial power factor after 1000 bending cycles. The intrinsically low thermal conductivity of PVP and its large number of nano/micro‐pores, as well as the effect of PVP/Ag_2_Se hetero‐interfaces (which can scatter phonons with short to long wavelengths) (Figure 12b) resulted in a low thermal conductivity for the PVP/Ag_2_Se composite films. A *zT* value of 1.1 at 300 K was obtained, an increase of 37.5% relative to the pristine Ag_2_Se film in the absence of PVP. Likewise, a PANI‐coated Ag_2_Se nanowires/PVDF composite film displayed a maximum power factor of 196.6 µW m^−1^ K^−2^ at 300 K with 65 wt% Ag_2_Se nanowires.^[^ ^135^ ^]^ Upon the assembly of a thermoelectric device with six legs of PVDF‐based composite film, an output voltage of 15.4 mV and a power of 835.8 nW were generated at a temperature difference of 30 K.

An Ag_2_Se/Ag/PEDOT composite film on a nylon membrane was engineered, which demonstrated a power factor of ≈1442.5 µW m^−1^ K^−2^, an electrical conductivity of ≈5957.3 S cm^−1^ and a good flexibility.^[^ ^138^ ^]^ A 5.5% decline in electrical conductivity after 1000 bending cycles at room temperature was observed. The high electrical conductivity of the composite film was ascribed to the presence of an Ag phase, while the low thermal conductivity arose from PEDOT at the interface between Ag and Ag_2_Se grains, and at the surface of Ag_2_Se grains. The excellent flexibility was credited to the synergistic effects of the PEDOT layer and the nylon membrane, which enhanced the bonding between inorganic phases and the binding of the composite film onto the nylon.

An Ag_2_Se/Se/PPy composite film was fabricated by the in situ polymerization of PPy at the surface of Ag_2_Se nanostructures, and showed outstanding thermoelectric properties at room temperature.^[^ ^134^ ^]^ The composite film exhibited dense microstructures with nano‐ to sub‐micrometer pores, which resulted from the in situ polymerization of PPy nanoshells. These nanoshells inhibit the sintering of Ag_2_Se nanostructures at lower temperatures. Moreover, the well‐developed crystalline Ag_2_Se grains afforded the material a high electrical conductivity. Despite a lower Seebeck coefficient than was expected from the n‐type Ag_2_Se and p‐type PPy, the energy‐filtering effects at the heterointerfaces of Ag_2_Se/Se and Ag_2_Se/PPy in the film yielded a positive effect on the Seebeck coefficient, leading to a high‐power factor of ≈2240 µW m^−1^ K^−2^ at 300 K. The nano‐ to sub‐micrometer pores and heterointerfaces in the composite film were able to scatter short‐ to long‐wavelength phonons, leading to low thermal conductivity. These attributes resulted in a high *zT* value of 0.94 at 300 K, which was a 15% enhancement compared to that of the pristine Ag_2_Se film without PPy. The Ag_2_Se/Se/PPy composite film exhibited a decline of 6.5% in electrical conductivity after 1000 bending cycles.

## 9 Emerging Thermoelectric Applications

Typically, thermoelectric generators are based on the principle of the Seebeck effect and consist of dissimilar thermocouples (i.e., p‐type and n‐type semiconductors), which are connected electrically in series and thermally in parallel. With design simplicity, no moving parts, free maintenance, long lifetime and environmental friendliness without toxic byproducts, thermoelectric generators have become an attractive proposition in energy harvesting for a wide variety of applications in automobile engines,^[^ ^196^, ^197^, ^198^, ^199^, ^200^, ^201^ ^]^ self‐powered electronic devices,^[^ ^202^, ^203^, ^204^, ^205^ ^]^ health monitoring and tracking systems,^[^ ^206^, ^207^, ^208^ ^]^ and aerospace systems.^[^ ^209^, ^210^ ^]^ Various sizes of thermoelectric generators are needed to meet the demands of these applications, from large to micro‐generators, which supply output powers ranging from several hundreds of watts to milliwatts across a vast range of temperature.^[^ ^211^, ^212^ ^]^

The Paris Climate Agreement on energy aims to substantially lower greenhouse gas emissions by at least 20% by 2030, compared to 1990 levels. The major sources of greenhouse gas emissions are power generation, manufacturing processes and transportation.^[^ ^213^ ^]^ These activities generate numerous waste heat sources at low (<250 °C), medium (250−650 °C) and high (>650 °C) temperatures. In order to utilize this waste heat for appreciable power generation, thermoelectric generators have mostly been proposed as waste‐heat recovery systems, capable of converting thermal energy into a useful DC power source.^[^ ^30^ ^]^

### 9.1 High‐Temperature Industrial Waste Heat Recovery/Conversion to Electric Power

A high proportion of the 33% of industrial energy used in manufacturing is dissipated directly into the atmosphere as industrial waste heat, which is sufficient to generate 0.9−2.8 TWh of clean energy per year via thermoelectric modules.^[^ ^214^ ^]^ An electrical output of ≈214 W could be generated from high‐temperature waste heat, using DC/AC inverters and a thermoelectric system attached to a carburizing furnace.^[^ ^215^ ^]^ The energy harvested in this case could be used to power the factory housing the furnace, thereby greatly increasing its efficiency.

Energy‐intensive steelmaking generates enormous quantities of waste heat, especially radiant heat from steel products. This heat can produce power thermoelectrically with an output of approximately 9 kW when continuously casting slab at ≈1188 K.^[^ ^216^, ^217^ ^]^ Likewise, energy‐intensive cement manufacturing consumes a great amount of energy (3.2−6.3 GJ) per ton of clinker generated.^[^ ^218^ ^]^ About 10–15% of the energy is lost as heat during the process. In this case, this energy could theoretically produce ≈211 kW of electrical power, whilst saving 3283 kW of energy.^[^ ^219^ ^]^ Glass manufacturing processes involve the melting of glass pellets above 1500 °C. These processes could thermoelectrically generate 55.6 kW of electricity from the production of 500 tons of glass per day, based on a typical *zT* value of ≈1. This technology can recover up to 1.37 billion kWh of electricity annually throughout the glass processing factories in the United States, which is equivalent to annual savings of $112 million in the energy cost (or 7.71 × 10^6^ kg of equivalent CO_2_ emissions) for the production of 20 million tons of glass per year.^[^ ^220^ ^]^

### 9.2 Medium‐Temperature Automotive Waste Heat Recovery to Electric Power

Automotive industries are projecting a burgeoning interest in thermoelectric generators for turning the exhaust gas waste heat produced by internal combustion engines into electrical energy.^[^ ^198^ ^]^ In a typical gasoline engine vehicle, approximately 20–30% of the combusted fuel is converted into useful energy for powering vehicles (about 30–45% for a diesel engine vehicle),^[^ ^221^, ^222^ ^]^ whereas the rest of the energy is lost as waste heat through the exhaust and cooling systems. Considering 6% of the exhaust waste heat could be converted into electrical energy, it would be possible to lower fuel consumption by up to 10%.^[^ ^223^ ^]^ The study on waste energy recovery from exhaust gases in a diesel passenger car revealed the potential fuel savings ranged from 8% to 19%.^[^ ^224^ ^]^

Major automobile manufacturers such as BMW,^[^ ^225^ ^]^ Renault,^[^ ^226^ ^]^ Honda,^[^ ^227^ ^]^ and Ford^[^ ^228^ ^]^ have started investing in thermoelectric generator‐based waste heat harvesting systems for their new‐generation vehicles. In 2013, Fiat and Chrysler announced the first commercial vehicle equipped with a thermoelectric generator, which achieved a 4% fuel economy improvement by harvesting electrical energy from its exhaust system.^[^ ^229^ ^]^ Particularly, segmented thermoelectric materials such as TAGS [(AgSbTe_2_)_1‐_ *_x_* (GeTe)*_x_*] have been used in the industry, due to their wide operational temperature range.^[^ ^230^ ^]^

AgSbTe_2_ is well known for its good thermoelectric properties in the medium temperature range (250–500 °C), as it possesses a large Seebeck coefficient and a low thermal conductivity.^[^ ^171^, ^231^, ^232^, ^233^, ^234^ ^]^ The excellent thermoelectric performance of AgSbTe_2_ indicates a great potential for usage in waste heat recovery applications in automotive exhausts. A high *zT* value of 1.59 was delivered at 673K for AgSbTe_2_, due to its low thermal conductivity (0.3 W m^−1^ K^−1^).^[^ ^235^ ^]^ By optimizing the electronic transport via modulation of the disorder‐induced localized electronic states, and simultaneous suppression of the lattice thermal conductivity (due to the formation of cation‐ordered nanoscale domains), a maximum *zT* value of 2.6 was achieved in Cd‐doped AgSbTe_2_ at 573 K.^[^ ^106^ ^]^ When substituting Sb with Zn in AgSbTe_2_, the formation of intrinsic Ag_2_Te impurity phases was suppressed, which improved the thermal and mechanical stability, leading to a *zT* value of 1.9 at 584 K and a hardness value of ≈6.3 GPa for AgSb_0.96_Zn_0.04_Te_2_.^[^ ^112^ ^]^ Upon alloying with GeTe, p‐type (AgSbTe_2_)_0.15_(GeTe)_0.85_ exhibited a *zT* value of 1.65 at 727 K with 2% neodymium doping.^[^ ^236^ ^]^ Notably, melt‐grown AgPb*_m_* SbTe*_m_*_+2_ synthesized by combining AgSbTe_2_ and PbTe was reported to exhibit a very high *zT* of 2.2 at 800 K.^[^ ^237^, ^238^ ^]^

### 9.3 Low‐Temperature Body Heat Recovery to Electric Power

Generally, a human body generates ≈100 W of heat at rest and ≈525 W during physical activity.^[^ ^239^ ^]^ Even with low‐efficiency thermoelectric generators, the constant heat energy from the human body is still sufficient to be collected and converted into electrical power for driving small devices like wearable electronics. Flexible thermoelectric generators in wearable electronics should possess various features, including i) high thermoelectric performance at near room temperature, ii) compliant mechanical flexibility for curved or irregular skins, iii) high stretchability to accommodate strains induced by body motions, and iv) self‐rescuing capabilities to heal mechanical damages in practical operation.

**Figure 13:**
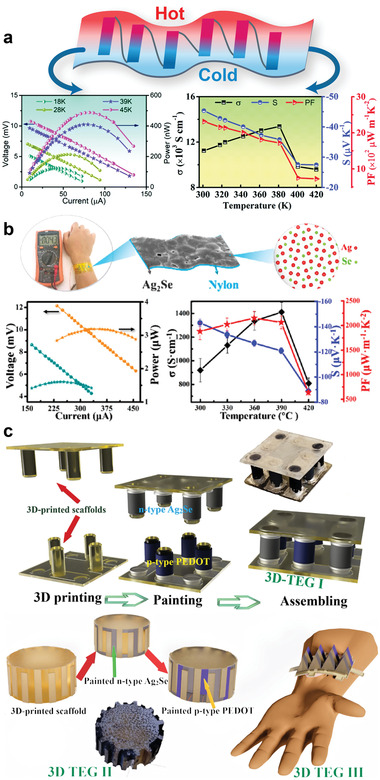
a) Schematic illustration of six‐leg TEG and thermoelectric performance of TEG fabricated with the flexible Ag_2_Se/Ag/CuAgSe/nylon composite films. Reproduced with permission.^[^ ^132^ ^]^ Copyright 2020, Royal Society of Chemistry. b) Schematic illustration and thermoelectric performance of the TEG fabricated with the flexible Ag_2_Se/nylon film. Digital photo of 4.3 mV voltage produced from four‐leg Ag_2_Se/nylon‐based TEG from a temperature difference of 6.7 K between the wrist and the environment. Reproduced with permission.^[^ ^129^ ^]^ Copyright 2020, American Chemical Society. c) Schematic illustration of the additive manufacturing‐based fabrication process: printing−painting−sintering and device structures of three different 3D‐TEG prototypes of cuboid shape, cylindrical gear shape and sawtooth shape. Reproduced with permission.^[^ ^143^ ^]^ Copyright 2022, American Chemical Society.

Jao et al. prepared a flexible thermoelectric generator (for use in a self‐powered temperature sensor) by the physical mixing of Ag_2_Te nanowires with conductive PEDOT:PSS,^[^ ^148^ ^]^ which demonstrated a Seebeck coefficient of 100 µV K^−1^ and an output voltage of 2.6 mV at a temperature difference of 25 K. A recent study reported a self‐powered flexible electronic device featuring a Ag_2_Te nanowire film,^[^ ^145^ ^]^ which generated a stable output voltage of ≈0.52 mV at room temperature, as the contact resistance between Ag_2_Te nanowires had been decreased by welding. A flexible thermoelectric module was assembled from four series‐connecting four‐leg Ag_2_Te films and generated ≈4 mV at a temperature difference of 40 °C. He et al. prepared a flexible thermoelectric generator by depositing Ag_2_Se nanowires on a flexible nylon membrane, from which a four‐leg assembly of binary Ag_2_Se/nylon composite film was created, yielding a maximum power density of 2.3 W m^−2^ at a temperature difference of 30 K. With the incorporation of Cu^+^, a power generator consisting of a six‐leg flexible Ag_2_Se/Ag/AgCuSe/nylon composite film produced an output voltage of 12.2 mV and a power density 5.42 W m^−2^ at a temperature difference of 45 K (**Figure** **13**a).^[^ ^132^ ^]^ A six‐leg flexible thermoelectric generator featuring a Ag/Ag_2_Se nano‐dendrites/nylon composite generated a voltage of 16.1 mV and a maximum power density of 13.56 W m^−2^ at a temperature difference of 29.6 K.^[^ ^131^ ^]^ Furthermore, the additional Ag phase in an eight‐leg thermoelectric prototype with a Ag/Ag_2_Se/nylon composite film generated a maximum power density of 8.74 W m^−2^ at a temperature difference of 27 K.^[^ ^130^ ^]^

A four‐leg thermoelectric generator was produced by assembling ternary Ag_2_Se/Ag/PEDOT composite films on a nylon membrane, which produced an output voltage of 5.6 mV and a power density of ≈7.47 W m^−2^ at a temperature difference of 27 K.^[^ ^138^ ^]^ A Ag_2_Se/PVP composite film assembled into a six‐leg thermoelectric generator produced a maximum power of 4.16 µW and a maximum power density of 28.8 W m^−2^ at a temperature difference of 29.1 K.^[^ ^133^ ^]^ This work demonstrated that an insulating polymer could act as a suitable additive for improving both the thermoelectric properties and the flexibility of inorganic thermoelectric films.

A flexible thermoelectric generator composed of six legs of ternary Ag_2_Se/Se/PPy composite films generated a voltage of 21.2 mV and a maximum power density of 37.6 W m^−2^ at a temperature difference of 34.1 K.^[^ ^134^ ^]^ Additionally, this flexible thermoelectric generator was successfully used to transform environmental low‐grade waste heat into electricity. When the Ag_2_Se/Se/PPy composite‐based thermoelectric generator was put under a warm cell phone that just ended running a game program, an output voltage of 5.3 mV was generated from a temperature difference of ≈9.4 K between the cell phone and the ambient surroundings. This study also demonstrated the feasibility of preparing flexible composite films for applications in wearable electronics.

In order to form a device capable of harvesting human body heat, a four‐leg thermoelectric generator was fabricated from Ag_2_Se/nylon composite films.^[^ ^129^ ^]^ The high density of Ag_2_Se films and the tuned orientation of grains (by multisized Ag_2_Se nanostructures) were expected to result in excellent thermoelectric performance. As a means to generate electricity from the temperature difference between the skin and the environment, one side of the thermoelectric generator was worn on the wrist and the other side was separated from the skin using a bubble film as a thermal insulator. The device demonstrated a maximum output power of 3.2 µW at a temperature difference of 30 K, corresponding to a maximum power density of 22.0 W m^−2^ and a normalized maximum power density of 408 µW m^−1^ K^−2^ (Figure 13b). In comparison with the room‐temperature thermoelectric properties of the flexible Ag_2_Se hybrid films reported above, this value is almost ten times as high as that of the four‐leg Ag_2_Se/nylon device (2.3 W m^−2^) and four times as high as that of the six‐leg Ag_2_Se/Ag/AgCuSe/nylon device (5.42 W m^−2^) at the same temperature difference of 30 K.

Ductile semiconductors present a different approach for developing portable and sustainable flexible thermoelectrics.^[^ ^182^ ^]^ A series of room‐temperature p‐type ductile thermoelectric semiconductors, AgCu(Se,S,Te) pseudoternary solid solutions, with a *zT* value of 0.45 at 300 K was fabricated into flexible *π*‐shaped thermoelectric devices with 0.3 mm in thickness.^[^ ^118^ ^]^ Specifically, p‐type (AgCu)_0.998_Se_0.22_S_0.08_Te_0.7_ was coupled with n‐type Ag_20_S_7_Te_3_ (connected electrically in series and thermally in parallel) between two polyimide‐based flexible circuit boards. The six‐couple (AgCu)_0.998_Se_0.22_S_0.08_Te_0.7_/Ag_20_S_7_Te_3_ legs produced an open circuit voltage of 3.7 mV and a maximum output power of 203 mW at a temperature difference of 1.5 K, generating a maximum normalized power density of up to 30 mW cm^−2^ K^−2^. This value is approximately four times higher than that of Bi_2_Te_3_‐based thermoelectric generators^[^ ^240^, ^241^, ^242^ ^]^ and 10000 times greater than that of organic‐based flexible thermoelectric generators.^[^ ^243^, ^244^, ^245^ ^]^ A cross‐plane *π*‐shaped thermoelectric device with 31‐couple (AgCu)_0.998_Se_0.22_S_0.08_Te_0.7_/Ag_20_S_7_Te_3_ was fabricated that effectively adhered to the curved surface of human skin. Under an ambient temperature of 298 K and when attached to a human's wrist, the device produced an open circuit voltage of 0.2 mV and maximum output power of 70 nW. The maximum normalized power density of this device was ≈11 mW cm^−2^ K^−2^.

Flexible thermoelectric components with complex shapes may be fabricated by 3D printing with additive manufacturing technologies. Thermoelectric inks are prepared by embedding the thermoelectric material in a passive matrix of additives and solvents. The resulting composite can then be printed by screen or ink‐jet printing onto mechanically flexible substrates, which is typically followed by a sintering process.^[^ ^142^, ^246^, ^247^ ^]^ For instance, a UV‑cured Ag_2_Se‐based thermoelectric composite was fabricated using a 3D printer based on digital‐light‐processing techniques, achieving a power factor of ≈51.5 µW m^−1^ K^−2^ at room temperature.^[^ ^31^ ^]^ Mallick et al. reported a one‐pot synthesis and processing of n‐type Ag_2_Se‐based screen‐printed materials with mixed Ag and Se powder.^[^ ^142^ ^]^ The targeted orthorhombic *β*‐Ag_2_Se phase was formed via postprinting sintering at moderate temperature, thereby reducing the detrimental impacts of binder and solvent. A volatilized phase of Se was adsorbed dissociatively by Ag to create Ag_2_Se without defined grain boundaries and a high conductivity transport path. Consequently, the printed material exhibited a power factor of 17 m^−1^ K^−2^ and a *zT* value of ≈1 at room temperature, which was significantly higher than the *zT* of 0.6 for a printed Ag_2_Se film with silver paste and Se particle ink.^[^ ^141^ ^]^ Furthermore, a printed thermoelectric generator with two thermocouples (Ag_2_Se‐based material as the n‐type leg and PEDOT:PSS as the p‐type leg), achieved an output voltage of 17.6 mV with a high maximum power output of 0.19 µW at a temperature difference of 60 K.

Three flexible and shape‐versatile thermoelectric generator prototypes (cuboid, cylindrical gear, and sawtooth) were successfully printed by stereolithography, a form of 3D printing, while an Ag_2_Se‐based ink was prepared based on the stoichiometric ratio of Ag and Se powders in solvent and additive.^[^ ^143^ ^]^ 3D‐thermoelectric generators were then fabricated using Ag_2_Se‐based ink as n‐type legs and PEDOT as p‐type legs on the surface of 3D‐printed resin scaffolds of different shapes. The thermoelectric legs were connected electrically in series and thermally in parallel. The printed thermoelectric generators were then sintered to yield a *β*‐Ag_2_Se phase in the n‐type legs (Figure 13c). As for the thermoelectric performance, maximum power outputs of ≈0.4 µW or 7 µW were generated by the cuboid (4 thermocouples) and cylindrical gear (9 thermocouples) thermoelectric generators respectively, at a temperature difference of 70 K. Upon introducing the system to the skin, an open‐circuit voltage of 4.2 mV at room temperature was achieved by the sawtooth‐shaped 3D thermoelectric generator (8 thermocouples).

## 10 Conclusions and Perspective

This review outlines the recent achievements of emerging silver‐based chalcogenides as thermoelectric materials in binary, ternary, quaternary, quinary, senary, and septenary compositions, which are prepared through various reactions, alloying, and doping processes with different chalcogens and other elements such as Cu, Au, In, Sn, Sb, and Bi. Significant progress in the development of thermoelectric materials in the form of bulk solids, films, nanoparticles/nanowires, and hybrids has been made across a wide temperature range, from room temperature to 900 K. The wide temperature applicability of silver‐based chalcogenide thermoelectric materials is an important factor in enabling the effective harvesting of heat at different scales. For example, large quantities of heat are encountered in industrial settings, such as within the steel production industry and power stations, whilst intermediate levels of heat are generated in exhausts and alternators of cars, and microscale heat is created by the human body.

Silver‐based chalcogenides (including those which are doped, alloyed, hybridized, and composited) have been considered in the nascent stages of development for many thermoelectric materials, but a number of challenges have been encountered so far. For instance, except for few of the widely studied silver‐based chalcogenides, the synthesis of many of the multinary silver chalcogenides in a phase‐pure form remains challenging, which hampers the study of the thermoelectric behavior of these materials. Often, the existence of a number of metastable phases leads to performance instability and irreproducibility. Such thermoelectric performance irreproducibility was frequently reported in liquid‐like thermoelectric materials, likely due to the migration of liquid‐like Ag atoms upon external stimulation (e.g., electric field or temperature gradient), which typically led to severe reductions in performance. Moving forward, ensuring that materials possess good stability and high *zT* under operating conditions is of vital importance for bridging the gap between fundamental materials science and practical applications, and for advancing thermoelectric technology towards commercial maturity.

Aside from those already studied, there remains a vast number of silver chalcogenide semiconductors, especially multinary compounds, that are yet to be examined for their thermoelectric potential. In the same way, sulfide counterparts also warrant more attention, as the majority of multinary compounds studied thus far are those of selenides and tellurides. Moreover, for a wider adoption of silver‐based chalcogenides in thermoelectric applications, such materials are expected to acquire crystal behavior with respect to electrical conduction, and glass behavior with regard to thermal conduction. Therefore, a wide variety of material designs have been developed to offer various avenues for fine‐tuning key electronic properties and thermal transport parameters. Herein, we have reviewed a number of exemplary studies on silver‐based chalcogenide thermoelectric materials which employ various engineering approaches, including quantum confinement, modulation doping, and energy filtering. These strategies seek to modify band structure and transport properties to either tune electrical conductivity and Seebeck coefficient independently, or increase them both simultaneously. Another fruitful strategy to increase *zT* is to minimize the lattice thermal conductivity. This may be achieved via phonon engineering approaches which enhance phonon scattering and decrease lattice thermal conductivity, due to the incorporation of nanostructured precipitates, grain boundaries, multinary alloying, elemental doping, and point defect loading. Moreover, the inclusion of a second phase in heterogeneous thermoelectric hybrids is another route to achieving a high *zT* value.

As estimated, a *zT* value of ≈3 is needed to realize practical thermoelectric applications and enable the large‐scale applicability of waste‐heat‐recovery technologies. So far, it remains incredibly challenging to reach this value or greater, though a promising Ag‐doped GeTe, together with Sb, Pb, and Bi (Ge_0.61_Ag_0.11_Sb_0.13_Pb_0.12_Bi_0.01_Te), has been demonstrated to achieve a high *zT* value of 2.7 at 750 K, and a high conversion efficiency of 13.3% at a temperature difference of 506 K, among the highest in the entire thermoelectric community. Particularly, a substantial enhancement in power factor is needed to raise *zT* values, depending on further reductions in thermal conductivity and increases in electron conductivity. Apart from displaying high‐quality thermoelectric properties, a good thermoelectric material should be scalable and cost‐effective to facilitate mass production. Such a material should be able to form dense compacts for device integration and possess high thermal stability for extended periods. Refining the quantitative understanding of the relationship between structure or composition and the properties of these materials is essential before they may be rationally designed and prepared.

The large‐scale applicability of waste‐heat‐recovery technologies also largely depends on the cost and environmental impact of thermoelectric materials, which are mainly based on alloys and compounds of bismuth, tellurium, and lead. Eventually, these elements should be replaced, due to their scarcity and toxicity. Driven by the growing demands of wearable and flexible electronic devices, there is an emerging research direction which focuses on methods to implement thermoelectric components into self‐powered wearable devices which harvest microscale heat from the body. In enabling the widespread adoption of flexible thermoelectric devices, silver‐based chalcogenide composites (including those which are film‐, polymer‐, and fiber‐based) have great potential, because these materials can directly generate power from a temperature difference between the human body and the surrounding environment. In the urgent search for emerging green energy technologies, it is expected that silver‐based chalcogenide thermoelectric materials will make a meaningful contribution to the minimization of fossil fuel usage in the near future. Silver‐based chalcogenide thermoelectric materials in doped, alloyed, hybridized, and composited forms have already been utilized for a vast range of niche applications, and efforts to translate this technology to mainstream applications are ongoing.

## Conflict of Interest

The authors declare no conflict of interest.
